# A New Nitrogen-Rich Energetic Material with So Many Tautomers

**DOI:** 10.1021/acs.cgd.6c00591

**Published:** 2026-06-26

**Authors:** Fatemeh Safari, Emmanuele Parisi, Emin Varghese, Santanu Chaudhuri, Nancy L. Ross, Carla Slebodnick, Steven D. Jacobsen, Carla Manfredi, Alessandro Landi, Andrea Peluso, Jennifer Heidrich, Thomas M. Klapötke, Russell J. Hemley, Roberto Centore

**Affiliations:** † Department of Physics, 14681 University of Illinois Chicago, Chicago, Illinois 60607, United States; ‡ Department of Applied Science and Technology, Politecnico of Turin, Corso Duca degli Abruzzi 24, Turin I-10129, Italy; § Department of Civil, Materials, and Environmental Engineering, 14681 University of Illinois Chicago, Chicago, Illinois 60607, United States; ∥ Department of Geosciences, 1757 Virginia Tech, Blacksburg, Virginia 24061, United States; a Department of Chemistry, 1757 Virginia Tech, Blacksburg, Virginia 24061, United States; ⊥ Department of Earth Science, University of Colorado Boulder, Boulder, Colorado 80309, United States; # Department of Chemical Sciences, 9307 University of Naples Federico II, via Cinthia, Naples I-80126, Italy; ∇ Dipartimento di Chimica e Biologia “Adolfo Zambelli”, 19028 Università di Salerno, via Giovanni Paolo II 132, Fisciano, Salerno I-84084, Italy; ○ Department of Chemistry, Energetic Materials Research, 9183 Ludwig-Maximilian University, Münich D-81377, Germany; ◆ Department of Chemistry and Department of Earth and Environmental Sciences, University of Illinois Chicago, Chicago, Illinois 60607, United States

## Abstract

A new nitrogen-rich triazolo-triazole compound, (3-(6-methyl-1H-[1,2,4]­triazolo­[4,3-*b*]­[1,2,4]­triazol-3-yl)-1H-1,2,4-triazol-5-amine), TTT1, has been prepared, and its acid–base and tautomeric behavior has been investigated. In the pH range of 0.3–12, TTT1 can accept up to two protons, forming a monocation and a dication, and can deliver one proton, forming a monoanion. The tautomeric behavior is particularly rich for the monocation, for which computational analysis predicts four different tautomers in a narrow energy range of 2 kcal/mol. Two of these tautomers (2H-7H-8H and 3H-7H-8H) have been isolated in salts of the monocation with suitable counterions (chloride, bromide, perchlorate). Surprisingly, the most stable predicted tautomer, 1H-3H-7H, has not been found in the four crystallized salts of the monocation. The energetic perchlorate salt of the monocation (3H-7H-8H tautomer) shows good thermal stability and good stability to impact, friction, and electric discharge. The packing of this compound shows the formation of H-bonded dimers with interactions between N8–H···N1. The crystal structure of this energetic salt was studied experimentally up to 2.8 GPa; no phase change or decomposition was observed.

## Graphical abstract

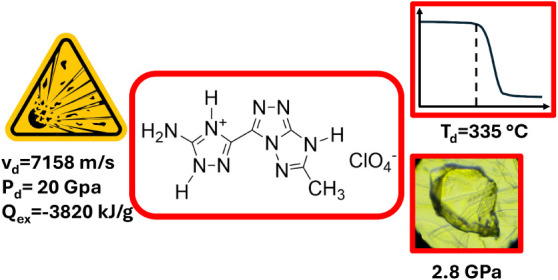

## Introduction

Fused-ring nitrogen-rich aromatics continue to be the focus of research on new high-energy-density materials (HEDMs).^1–3^ This interest arises from the endothermic nature of these molecules, which, in turn, is related to the high bond energy of the triple bond in N_2_ and the gas volume delivered upon decomposition. The rate of decomposition and the rate of volume released are key parameters in HEDMs.^4,5^

New high-energy-density molecules have been synthesized over the years; these N-rich energetic materials generally consist of an arrangement of C, N, H, and O, along with different combinations of backbones and functional groups, which determine properties such as density, intermolecular and intramolecular interactions, and crystal packing.^6–12^ These factors, in turn, have a profound impact on energy release and sensitivity.^13^ Since energetic materials can undergo phase transitions or decomposition reactions during detonation, studying their behavior under extreme conditions is essential for advancing their practical applications.^14–17^

In the field of N-rich aromatics for HEDMs, one relevant challenge is to increase the weight percent of nitrogen while keeping acceptable chemical and thermal stability and safe handling. One advantage of N-rich aromatics is the presence of basic N atoms, which can be exploited for the preparation of energetic salts with suitable oxidizing anions (perchlorate, nitrate, and dinitramide). N-rich aromatics can also have acidic N–H groups. In this case, the versatility of the N-rich compounds is further increased because of tautomerism^18–20^ and the possibility of preparing salts of the deprotonated anionic form with suitable cations.

Here, we present a full investigation, including high-pressure X-ray diffraction experiments, of the new N-rich heteroaromatic compound (3-(6-methyl-1H-[1,2,4]­triazolo­[4,3-*b*]­[1,2,4]­triazol-3-yl)-1H-1,2,4-triazol-5-amine), henceforth TTT1 (Chart [Fig chart1]), featuring a nitrogen content of 61.4% and showing both basic N atoms and acidic N–H groups. TTT1 belongs to the class of 1,2,4-triazole-based energetic materials, for which promising behavior has been reported in many cases.^1–3,21,22^

**1 chart1:**
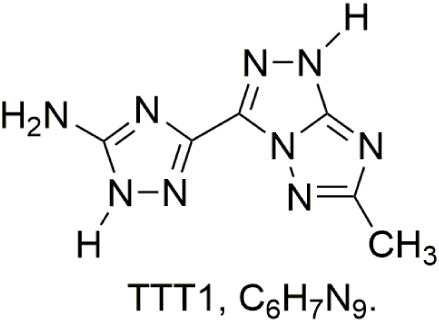
Chemical Diagram and Formula of TTT1

## Results and Discussion

### Tautomerism

The tautomeric behavior of TTT1 is summarized in Chart [Fig chart2], in which some of the canonical tautomeric forms expected for the neutral, singly protonated, doubly protonated, and singly deprotonated species are shown (a more complete list is reported in Supporting Information) with indication of the numbering of N atoms we have adopted. The computed relative energies of the tautomers (water as the continuum polarizable medium; see the Supporting Information for details) are also shown in Chart [Fig chart2].

**2 chart2:**
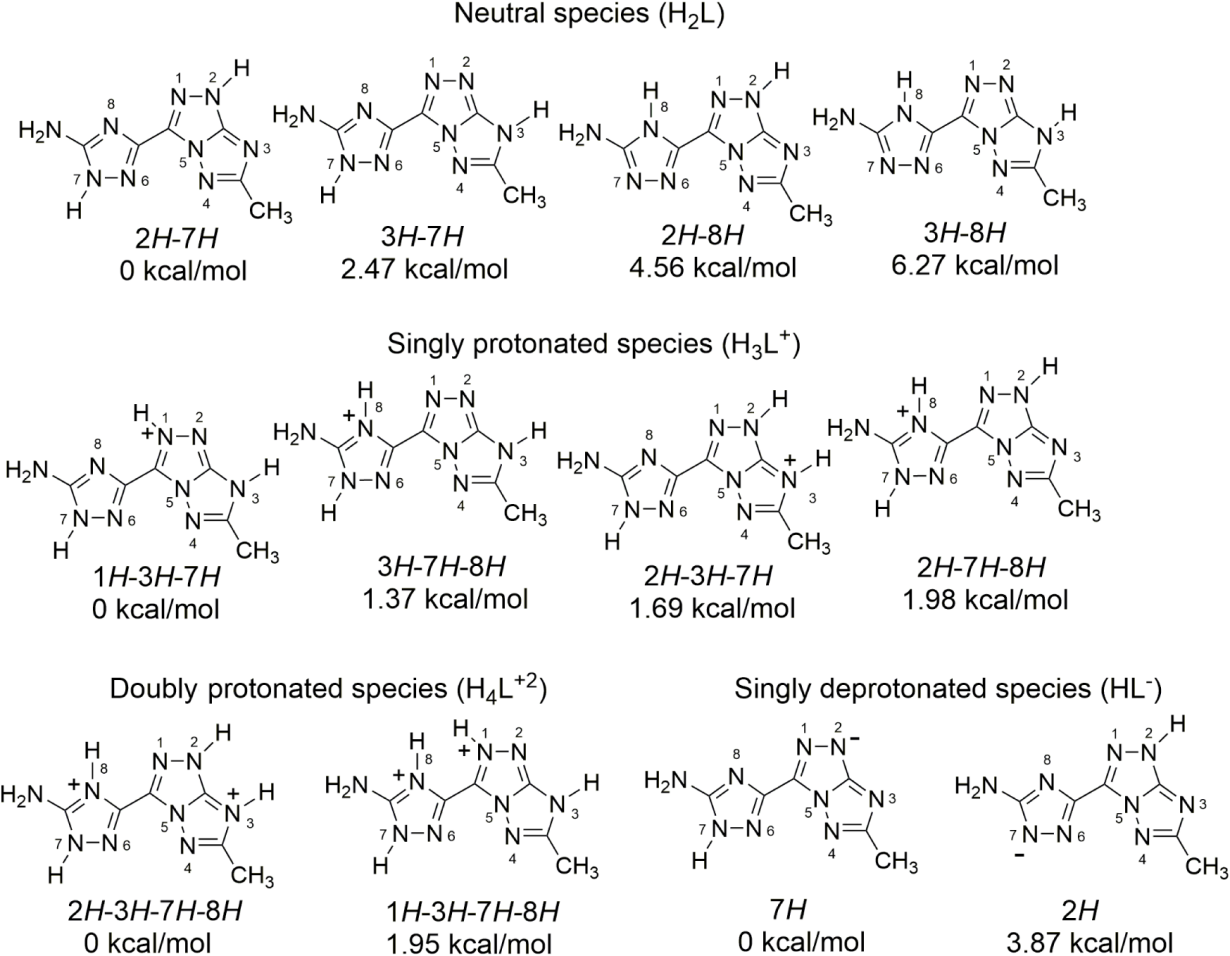
Some Tautomers of TTT1

For the neutral species, the computed relative energies of the tautomers follow the trend already observed in previous similar systems,^23–26^ with one tautomer, 2H-7H, being the most stable and the next one close in energy, 3H-7H, being 2.47 kcal/mol higher. The computed energies for the singly protonated species show a different pattern because, in this case, remarkably, there are four different tautomers within the small energy range of 2 kcal/mol. This would correspond to a Boltzmann population, at room temperature, of 84%, 8%, 5%, and 3%, respectively, for the four tautomers in chemical equilibrium with each other.

### Acid–Base Equilibria in Solution

The effective formation of cationic and anionic species of TTT1, as anticipated in Chart [Fig chart2], has been proven experimentally by spectrophotometric acid–base titrations in solution at constant concentration (see Supporting Information). In the investigated pH regime (0.3–12), three different protolytic equilibria were recognized, in which the dication, monocation, neutral, and monoanion species are involved. The calculated conditional equilibrium constants are reported in Table 1, in which the neutral TTT1 species is indicated by *H*_2_ *L*.

**Table 1:** Acid Constants in the Form of pK_a_ for TTT1 (***H***_2_ ***L***), with Estimated Standard Deviations^a^

Reaction	Equilibrium constant
$H_{4}L^{2+}+H_{2}O⇄H_{3}L^{+}+H_{3}O^{+}$	***pK***_**a**1_ = 0.6 ± 0.1
$H_{3}L^{+}+H_{2}O⇄H_{2}L+H_{3}O^{+}$	***pK*** **a**_2_ = 1.8 ± 0.1
$H_{2}L+H_{2}O⇄HL^{-}+H_{3}O^{+}$	***pK*_a3_** = 6.4 ± 0.1

a The distribution diagram is reported in Figure 1.

From Figure 1, it comes out that there exist definite pH intervals in which each of the four species involved in the protolytic equilibria is predominant in solution. This could allow salts containing each of the three ionic species of TTT1 to be isolated from the solutions.

**Figure 1:**
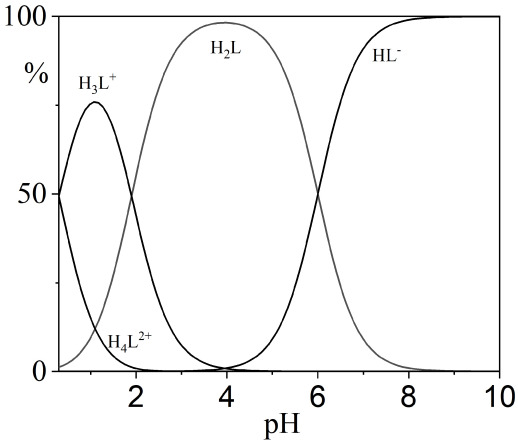
Distribution diagram of TTT1 evaluated using the equilibrium constants of Table 1.

### Solid-State Structural Analysis and Tautomer Identification

The versatility of TTT1 is suggested by the presence of many low-energy tautomers, in particular for the monocation *H*_3_ *L* ^+^, and by the three protolytic equilibria in an accessible pH range. So, starting from the sole TTT1, we can expect the formation of a lot of different products, differing in the protonation degree (because of the pH) and in the location of acidic H atoms (because of tautomerism). By reacting TTT1 with solutions of strong mineral acids or bases in suitable concentrations, we have prepared many salts that were studied by single-crystal X-ray analysis to check which tautomer was present. The results are summarized in Table 2. As expected, based on the energies reported in Chart [Fig chart2], variability in the tautomer that crystallizes is observed for the monoprotonated species. It is somewhat surprising, however, that the most stable predicted tautomer of *H*_3_ *L* ^+^, i.e., 1H-3H-7H, was not observed in the crystals. The two tautomers observed instead are 3H-7H-8H (+1.37 kcal/mol) and the least stable one (2H-7H-8H, +1.98 kcal/mol). As detailed in Supporting Information, the packing of these salts is mainly driven by strong H-bonds (N–H···N, N–H···O, N–H···X^–^) and by stacking interactions (N···N, C···N), as many of the salts form layered structures.

**Table 2:** Prepared Salts of TTT1 with Indication of the Species and Tautomer Present

Numbering	acid/base	Species	Tautomer
1	none	$H_{2}L$	2H-7H
2	HCl	***H***_2_ ***L***/***H***_3_ ***L*** ^+^	2H-7*H*/2H-7H-8H
3	HCl	$H_{3}L^{+}$	2H-7H-8H
4	HCl	$H_{4}L^{2+}$	2H-3H-7H-8H
5	HBr	$H_{3}L^{+}$	3H-7H-8H
6	HClO_4_	$H_{3}L^{+}$	3H-7H-8H
7	H_2_SO_4_	$H_{4}L^{2+}$	2H-3H-7H-8H
8	KOH	$HL^{-}$	7H

The X-ray molecular structures of TTT1 and various salts, obtained with hydrochloric acid, are shown in Figure 2.

**Figure 2:**
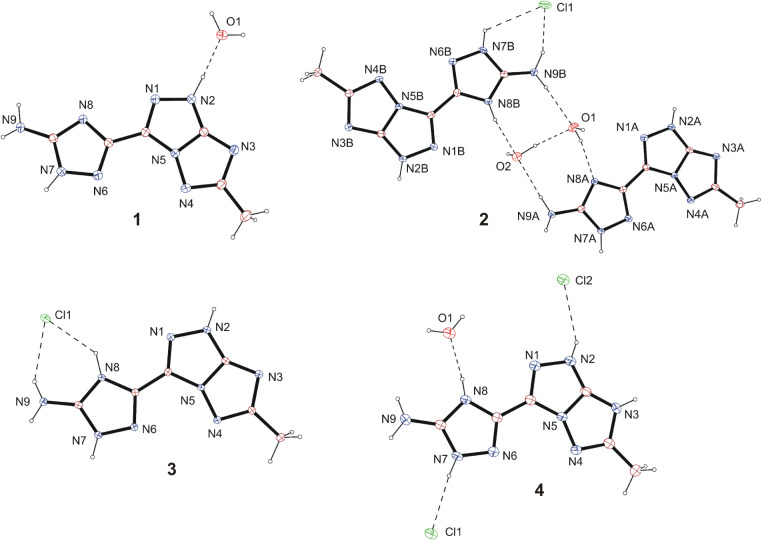
X-ray molecular structures of **1**, **2**, **3,** and **4**. Selected H-bonds are shown as dashed lines. For **4,** only one of the two crystallographically independent cation molecules is shown.

Neutral TTT1 crystallizes as a hydrate, compound **1**, and the most stable predicted 2H-7H tautomer is observed. With hydrochloric acid, at increasing concentrations of the acid, three different salts —**2**, **3**, and **4**—were obtained, as reported in Table 1 and Figure 2, at an increasing degree of protonation.

**2** is a cocrystal of the neutral and monoprotonated species. The neutral species is in the lowest-energy 2H-7H tautomer, while the monoprotonated species is in the highest-energy 2H-7H-8H tautomer. By increasing the concentration of HCl, salt **3** is obtained, in which only the monoprotonated species is present, again in the least stable 2H-7H-8H tautomer. At even higher HCl concentrations, salt **4** is obtained, in which the diprotonated species is present, in the most stable 2H-3H-7H-8H tautomer.

With hydrobromic acid, salt **5** is obtained (Figure 3), in which the monocation is present. Remarkably, and different from the analogous salt **3** with HCl, the tautomer 3H-7H-8H is found. The tautomer 3H-7H-8H of the monocation is also present in the perchlorate salt **6**, also shown in Figure 3, which, together with **3,** is the only one in Table 2 that crystallizes without water molecules in the unit cell.

**Figure 3:**
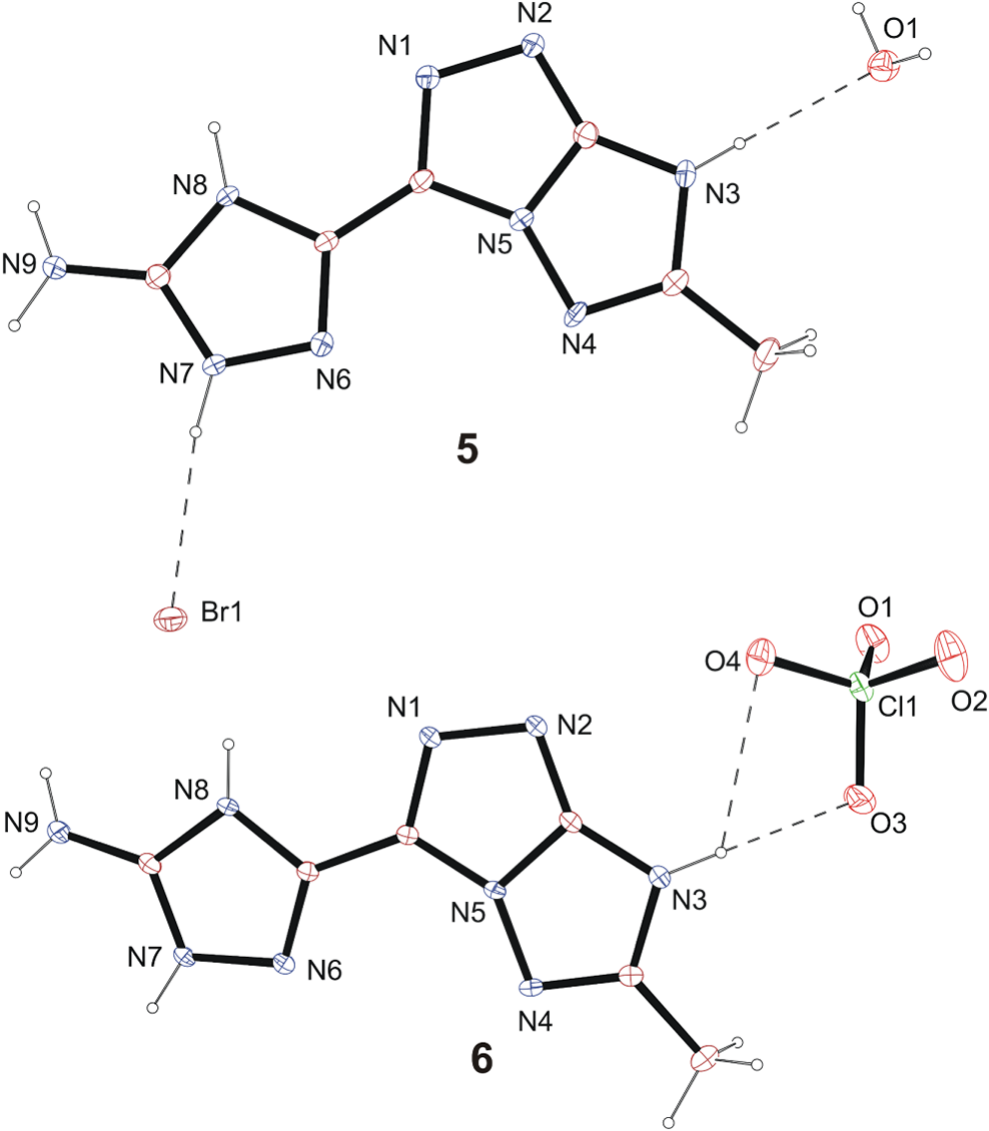
X-ray molecular structures of **5** and **6**. Selected H-bonds are shown as dashed lines.

With sulfuric acid, we were able to crystallize the dication, again as the most stable 2H-3H-7H-8H tautomer (Figure 4).

**Figure 4:**
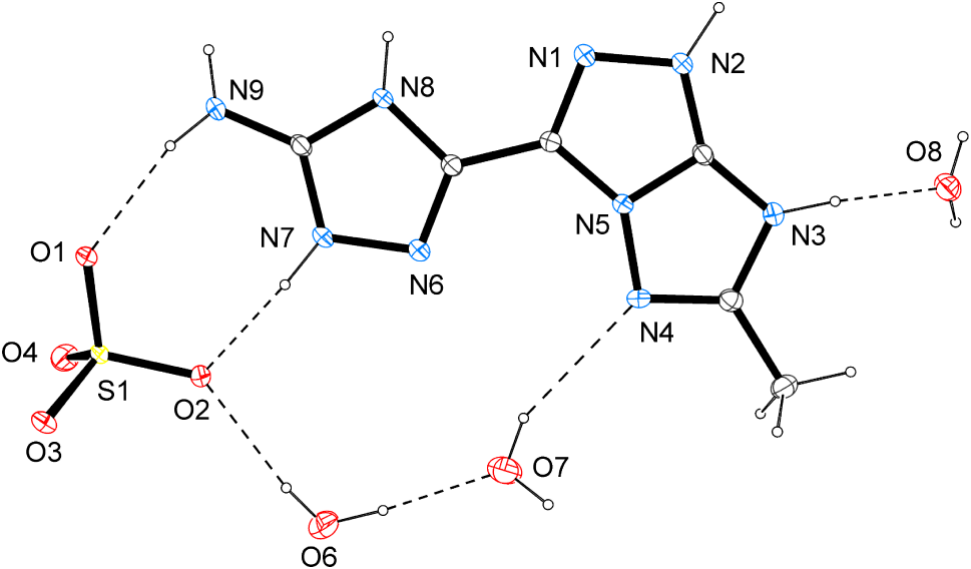
X-ray molecular structures of **7**. Selected H-bonds are shown as dashed lines.

Finally, the potassium salt of the monoanion was obtained by the treatment of TTT1 with aqueous KOH. The crystal structure analysis indicates that tautomer 7H is present in the salt (Figure 5). This is consistent with the expected higher acidity of N2–H with respect to that of N7–H. In fact, the negative charge on N2 of the anion can be delocalized, by resonance, onto N3, N4, and N6.

**Figure 5:**
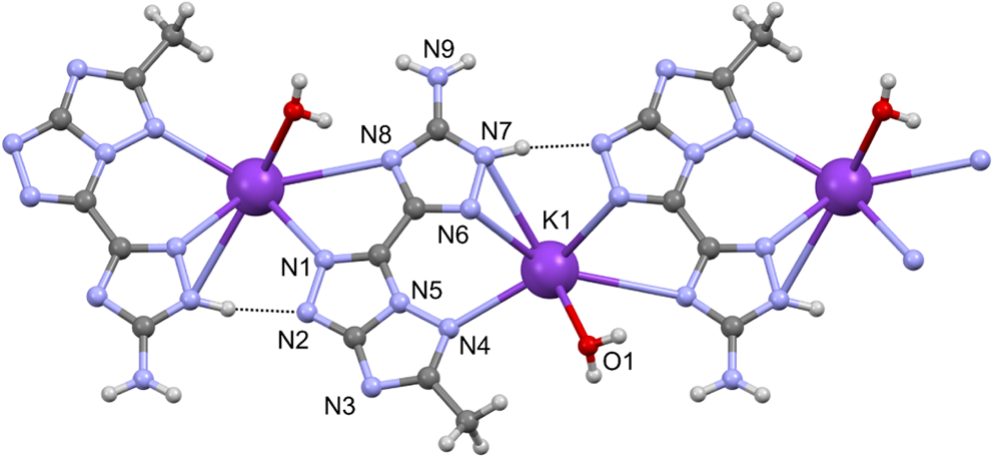
Partial packing of **8**. Selected H-bonds are shown as dashed lines.

The coordination at K^+^ is mainly due to steric and electrostatic factors because of its closed-shell electronic configuration. The triazolo-triazolate anion can act as a bidentate chelate ligand in two ways: (N1, N8), with the formation of a pentatomic chelate ring, and (N4, N6), in which the chelate ring is six-membered. Both ways are present in the crystal structure, Figure 5.

### The Energetic Perchlorate Salt 6 and the Elusive Most-Stable Tautomer 1H-3H-7H

The energetic perchlorate salt **6** was obtained by crystallization of TTT1 from 2 M perchloric acid (see the Experimental Part in the Supporting Information). The crystal structure of **6** is shown in Figure 6.

**Figure 6:**
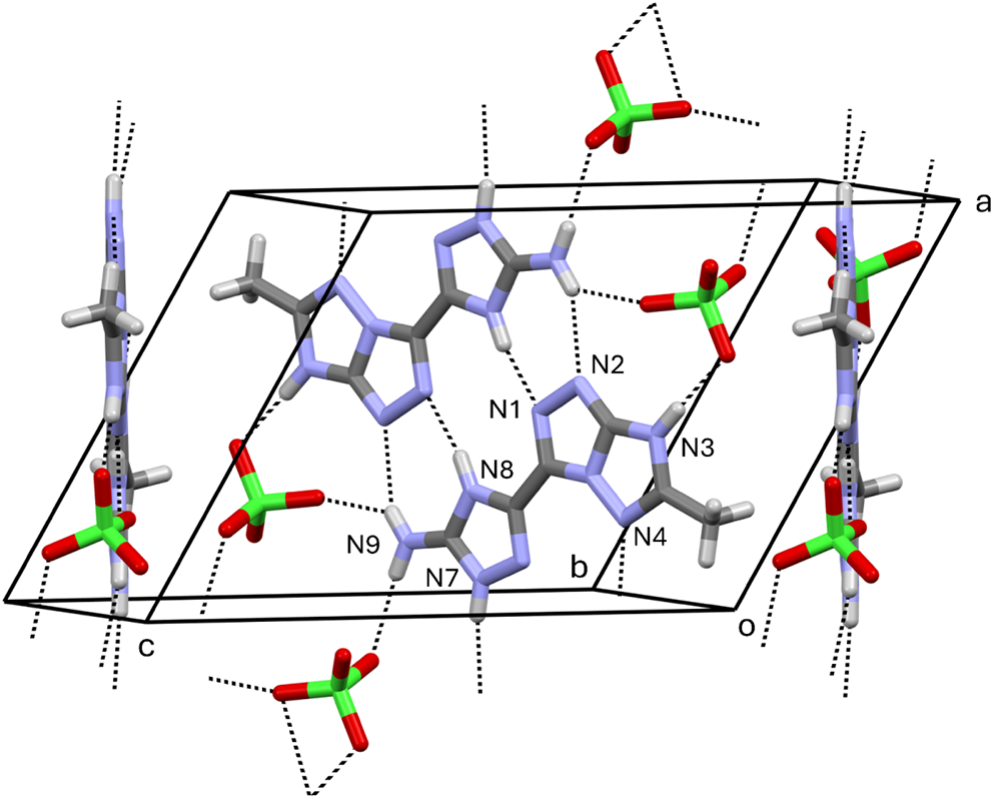
Crystal packing of **6**. H-bonds are shown by dashed lines.

The basic structural unit of the crystal packing is represented by a planar H-bonded dimer formed by two cations linked by several N–H···N hydrogen bonds. Each dimer is surrounded by four perchlorate anions, which are also involved in H-bonding with the N–H donors of the cations. The dimers are present in the crystal lattice with two different orientations. The energetic salt **6** has good thermal stability and good decomposition efficiency. As can be deduced from the combined TGA/DTA curves of Figure 7, **6** is stable up to 335 °C, and the decomposition is rather clean, spanning about 25 °C.

**Figure 7:**
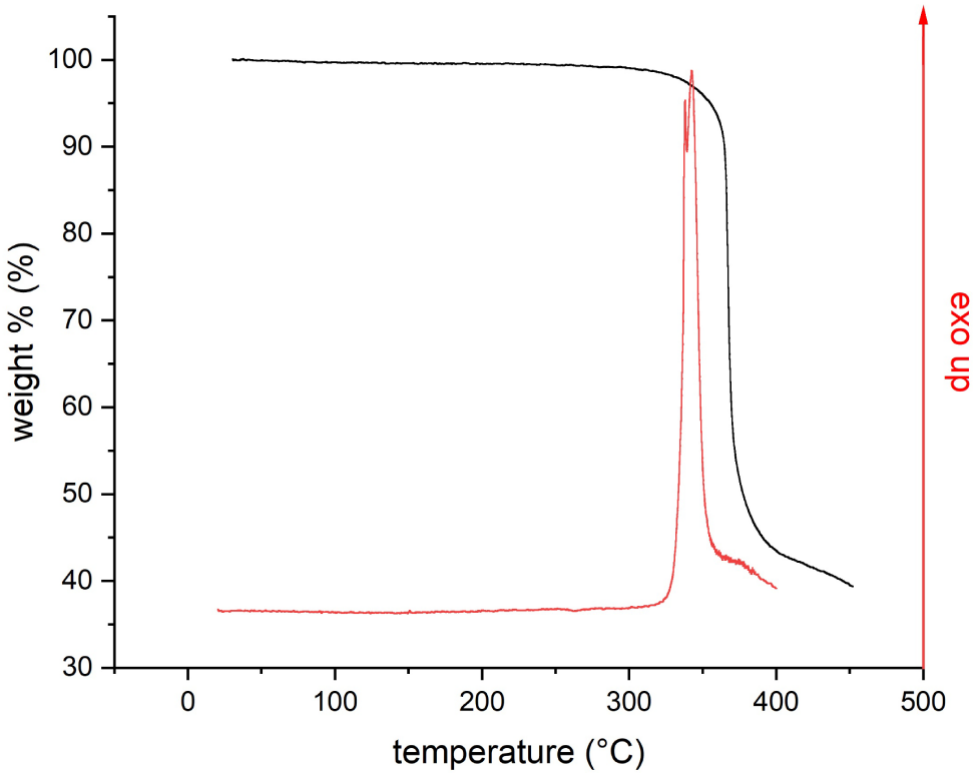
Combined TGA/DTA analysis of **6**. Heating rate: 5 °C/min. TGA was performed in flowing N_2_ and DTA in air.

Some energetic properties of **6** are reported in Table 3 and are compared with two well-known energetic materials, i.e., ammonium perchlorate (AP)^5^ and hexanitrostilbene (HNS).^4,5,27^ The impact (IS), friction (FS), and electrostatic discharge (ESD) sensitivities were measured experimentally (grain size: 100–500 μm).^28–32^ Based on the computed enthalpies of formation, the detonation parameters (velocity of detonation, detonation pressure, and heat of detonation) were calculated with the EXPLO5 program^33^ and are also reported in Table 3 (full details of calculations are given in Supporting Information).

**Table 3:** Energetic Properties and Detonation Parameters of **6** and Some Reference Compounds

	ρ (g/cm^3^)^a^	T_d_ (°C)^b^	Q_ex_ (kJ/g)^c^	Ω%^d^	P (GPa)^e^	V (m/s)^f^	IS (J)^g^	FS (N)^h^	ESD (J)^i^
6	1.69^j^	335	–3820	–60.2	19.6	7158	15	>360	1.0
AP	1.95	242		27.2	16	6520	20	>360	
HNS	1.74	318	–4008	–67.6	24.4	7270	5	>240	

a Density.

b Decomposition temperature.

c Heat of explosion.

d Oxygen balance based on CO_2_.

e Detonation pressure.

f Detonation velocity.

g Impact sensitivity.

h Friction sensitivity.

i Electrostatic discharge sensitivity.

j Density is calculated at room temperature (25 °C) from the crystallographic density at −100 °C (1.722 g/cm^3^) according to ref 4.

Data from Table 3 indicate that **6** can be classified as a thermally highly stable, moderate-energy new explosive with low sensitivity.^34^

The crystal structure of **6** shows that the tautomer 3H-7H-8H is present (Figures 3 and 6). As shown in Chart [Fig chart2], this tautomer is predicted to be 1.37 kcal/mol higher in energy as compared with the lowest energy one, 1H-3H-7H. In Figure 8a, the H-bonded dimer typical of the crystal structure of **6** is shown in more detail. It is clear that if the H atom bonded to N8 in the actual structure and involved in H bonding with N1 as the acceptor were to leap onto N1, a virtual structure would be formed (Figure 8b), in which the most stable 1H-3H-7H tautomer would be present.

**Figure 8:**
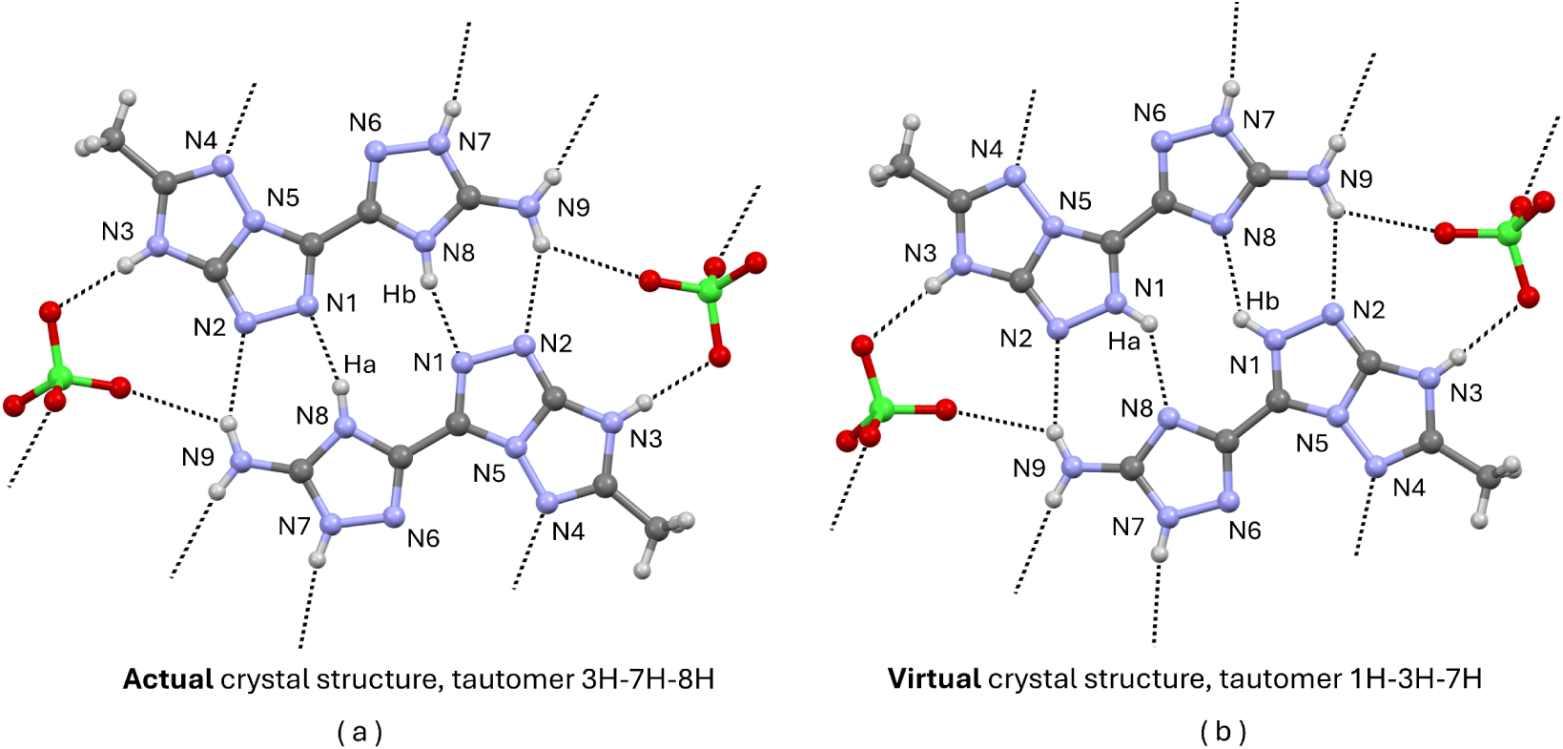
(a) A H-bonded dimer in the actual crystal structure of **6**; (b) the same H-bonded dimer of (a) in a hypothetical (virtual) structure in which the H atom bonded to N8 of (a) has been transferred onto N1 without any other change.

In this virtual structure, molecules would keep their positions, so the space group should not change, and small variations of the unit cell parameters are expected. The H bond N1···N8 would still be present, but with N8 as the acceptor. As a matter of fact, the two structures would only differ in the position of that H atom. Given that, at ambient pressure, the observed structure is that of Figure 8a both at room temperature and at −100 °C, we have considered the possibility of a tautomeric switching determined by pressure-induced proton transfer.^35^ So, we have undertaken a high-pressure single-crystal X-ray diffraction analysis of **6**.

### Measured Pressure Dependence of Unit Cell Parameters

Photomicrographs of the sample showed a well-defined, unchanged single-crystal habitus across the investigated pressure range (0.1 MPa–2.8 GPa; Figure 9).

**Figure 9:**
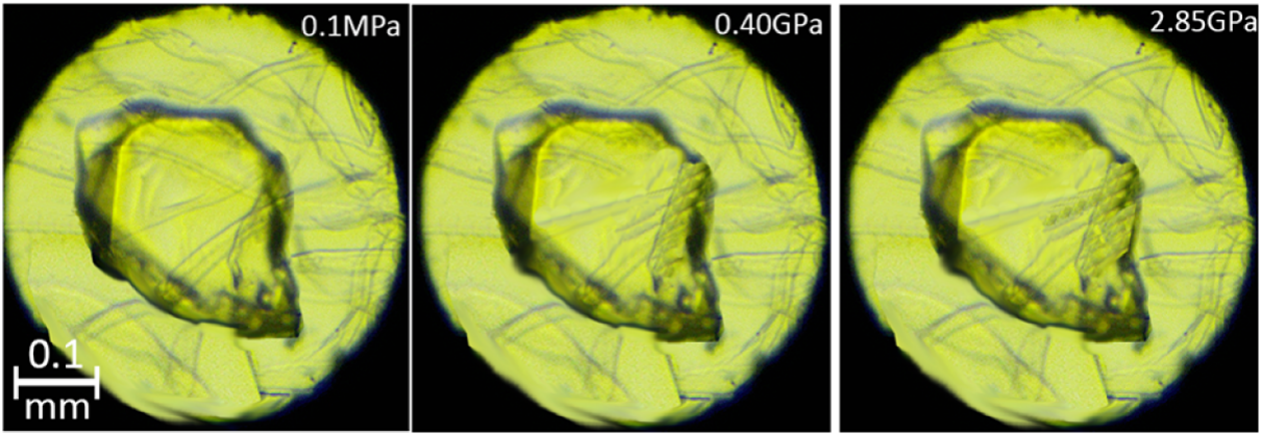
Photomicrographs of a single crystal of **6** compressed in Parabar 10312 in a diamond-anvil cell at 0.1 MPa, 0.40 GPa, and 2.8 GPa at 296 K. Small ruby chips were also placed in the sample chamber for pressure calibration.

The variation in the unit-cell parameters under pressure was measured up to 2.8 GPa by collecting X-ray diffraction data on a single crystal, as shown in Figure 10.

**Figure 10:**
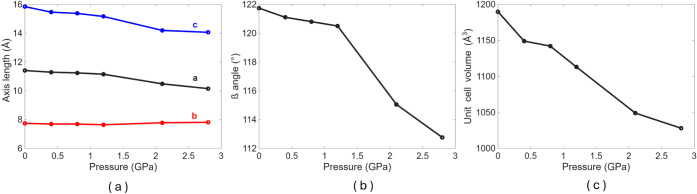
Experimental unit cell parameters of **6** at different pressures and room temperature. (a) Behavior of the unit cell axes; (b) behavior of the β angle of the monoclinic cell; (c) experimental unit cell volume of **6** at different pressures and room temperature.

The *a-* and *c*-axes undergo a contraction by increasing pressure, while the *b*-axis exhibits a comparatively minor but opposite variation, indicating that this lattice dimension is relatively less sensitive to pressure. The variation of the unit cell parameters is basically smooth in the range of pressure investigated, but for the β-angle, which shows a drop from about 120° to 112° above 1.2 GPa. The structural response of **6** to high pressure is thus anisotropic. In addition, a monotonic decrease in unit-cell volume with increasing pressure is consistent with compression behavior (Figure 10c).

### Computational Analysis of the Structures

Starting from the actual structure of **6** at 0.1 MPa, we have generated the virtual structure by simply shifting the H atom from N8 to N1, as shown in Figure 8. The two initial structures, actual and virtual, were used for computational analysis. In short, actual and virtual structures were optimized at different pressures by relaxing lattice parameters and fractional coordinates to determine their equilibrium volumes and lattice parameters.^36,37^ The pressures investigated were 0.1 MPa, 0.4 GPa, 0.6 GPa, 1.2 GPa, 2 GPa, 3 GPa, 4.3 GPa, 5.2 GPa, 6.4 GPa, 7.1 GPa, 8.4 GPa, and 9.3 GPa. The first result is that at each pressure, the actual structure is successfully optimized and corresponds to a minimum of lattice energy, that is, a stable structure. The virtual structure was successfully optimized up to ∼2.8 GPa. Systematic calculations at intermediate pressures between 2.0 and 3.0 GPa (see SI for further information) precisely identify this threshold, beyond which the optimization procedure spontaneously converts the virtual structure into the actual 3H-7H-8H structure, regardless of the starting configuration. The behavior of optimized cell parameters for actual and virtual structures with pressure is shown in Figure 11. Remarkably, the change of unit cell parameters with pressure for the optimized actual structures is like the experimental plots of Figure 10, including the drop of the β-angle, which is correctly predicted. Concerning the virtual structure in the range 0.1 MPa–2 GPa, some differences for *a*-axis and β-angle are observed. In Figure 11c, the predicted volume of the virtual structure at lower pressure is less than that of the actual structure. So, the virtual structure is predicted to have a higher density than the actual structure in the lower pressure regime. The enthalpies of actual and virtual structures were also calculated (Figure 12). The curves converge above ∼2.8 GPa, where the actual and virtual structures yield the identical 3H-7H-8H structure at these pressures. From ambient to 2 GPa, the virtual structure has a higher enthalpy than the actual one by 3.5–4.6 kcal/mol.

**Figure 11:**
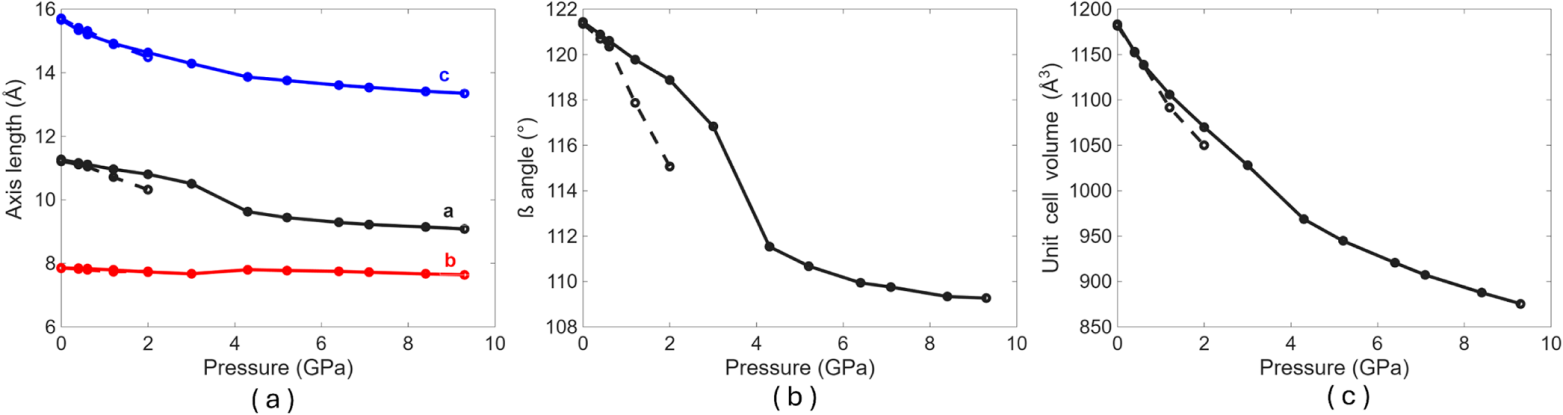
Optimized unit cell parameters and unit cell volume for actual (continuous lines) and virtual (dashed lines) structures of **6** at different pressures and room temperature. (a) Unit cell axes; (b) β angle of the monoclinic unit cell; (c) unit cell volume.

**Figure 12:**
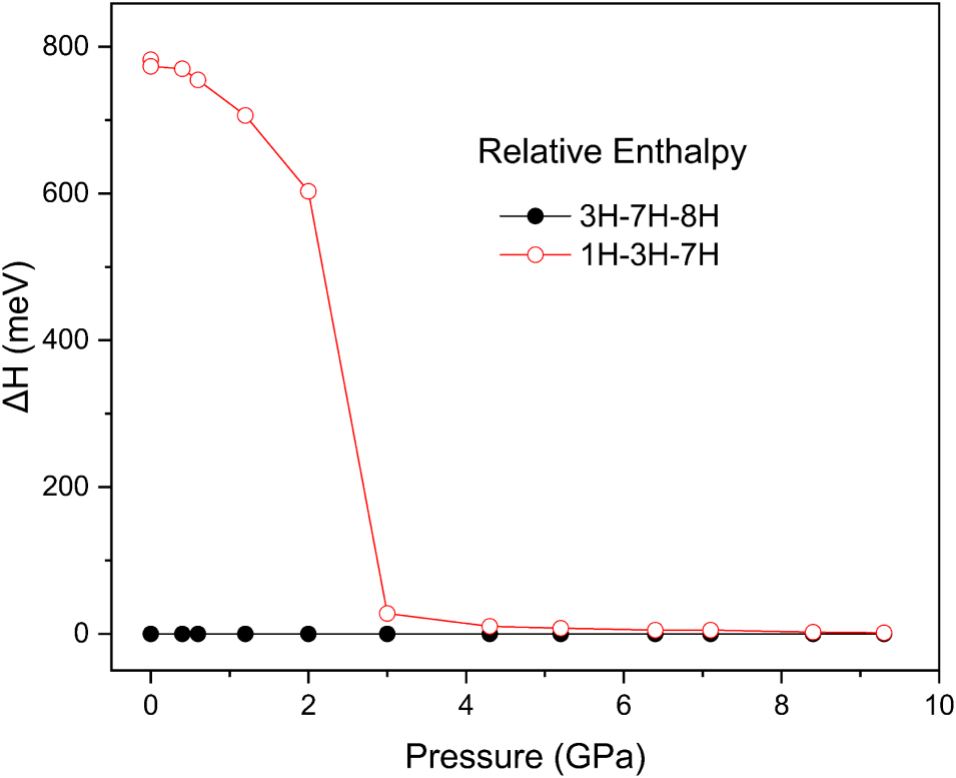
Calculated relative enthalpy between optimized actual (i.e., containing the 3H-7H-8H tautomer) and virtual (i.e., containing the 1H-3H-7H tautomer) crystal structures of **6** up to 10 GPa.

It remains to explain why the actual structure is more stable than the virtual one, notwithstanding its lower density and the presence of the higher-energy tautomer 3H-7H-8H. A possible hint may come from looking again at Figure 8, in particular, at the (repulsive) contact Ha···Hb. In the actual structure, containing the higher-energy 3H-7H-8H tautomer, the two H atoms are farther apart than in the virtual structure that contains the lower-energy 1H-3H-7H tautomer. By increasing pressure, those two H atoms get closer, regardless of whether the crystal structure is actual or virtual. In these conditions, it stands to reason that the close contact Ha···Hb becomes a strongly energetically penalizing feature of the virtual structure, which could only be released by deformation of bond angles, which are also energetically penalizing.

We also note that this picture is compatible with the hypothesis that, in the growth of crystals of **6** from solution, molecules of *H*_3_ *L* ^+^ cations enter the crystals (or the prenucleation clusters) in the most-abundant 1H-3H-7H tautomer, forming dimers like that of Figure 8b that quickly switch by proton transfer to the more stable dimer of Figure 8a without relevant changes to the growing crystal structure.

## Conclusions

We have prepared and studied a new nitrogen-rich high-energy-density compound. The study has revealed rich acid–base and tautomeric behaviors. For the monoprotonated species, four tautomers are predicted within 2 kcal/mol, and two were effectively identified. The tautomerism was further investigated by high-pressure single-crystal X-ray diffraction. The density functional theory calculations on energetic salt **6** reproduce key findings of the experimental high-pressure results and thus the subtle changes in intermolecular interactions induced by compression. DFT calculations under hydrostatic compression revealed that the experimentally observed crystal structure containing the 3H-7H-8H tautomer of the monocation remains mechanically stable throughout the investigated pressure range (0.1 MPa–9.3 GPa). In contrast, the virtual structure containing the 1H-3H-7H tautomer loses metastability above ∼2.8 GPa and spontaneously transforms into the 3H-7H-8H configuration during geometry optimization. This behavior indicates that compression destabilizes the 1H-3H-7H hydrogen-bonding network, causing the associated local energy minimum to disappear at elevated pressure. The results demonstrate that the 3H-7H-8H arrangement is the preferred high-pressure form and possesses greater structural robustness under compression.

## Supplementary Material



## Notes

Experimental part: Full description of the synthesis of TTT1 with ^1^H and ^13^C NMR spectra of the final compound and intermediates; description of the synthesis of salts; full description of the X-ray analysis; tables with full crystallographic details (crystal, collection, and refinement data); description of the packing of compounds **1**-**8**; analysis of Hirshfeld fingerprint plots; powder diffraction pattern of as-prepared samples of **6**; detailed description of the computational analysis of tautomers; detailed description of the computational analysis of actual and virtual structures of **6** at variable pressure (PDF)
