# Supplementary material for: A New Nitrogen-Rich Energetic Material with So Many Tautomers
Source: Cryst Growth Des. 2026 Jun 26;26(14):5648–56. doi: 10.1021/acs.cgd.6c00591 (PMC13383613; doi:10.1021/acs.cgd.6c00591)
Supplement: Supplementary file 1 [file cg6c00591_si_001.pdf]

Supporting information file

A New Nitrogen-Rich Energetic Material with So Many Tautomers

Fatemeh Safari,[a] Emmanuele Parisi,[b] Emin Varghese,[c] Santanu Chaudhuri,[c] Nancy L. Ross,[d] Carla Slebodnick,[e] Steven D. Jacobsen,[f] Carla Manfredi,[g] Alessandro Landi,[h] Andrea Peluso,[h] Jennifer Heidrich,[i] Thomas M. Klapötke,[i] Russell J. Hemley,[a,j] Roberto Centore[g]*

[a]Department of Physics, University of Illinois Chicago, Chicago, Illinois 60607, United States.
[b]Department of Applied Science and Technology, Politecnico of Turin, Turin I-10129, Italy.
[c]Department of Civil, Materials, and Environmental Engineering, University of Illinois Chicago, Chicago, Illinois 60607, United States.
[d]Department of Geosciences, Virginia Tech, Blacksburg, Virginia 24061, United States.
[e]Department of Chemistry, Virginia Tech, Blacksburg, Virginia 24061, United States.
[f]Department of Earth Science, University of Colorado Boulder, Boulder, Colorado 80309, United States.
[g]Department of Chemical Sciences, University of Naples Federico II, Naples I-80126, Italy.
[h]Dipartimento di Chimica e Biologia "Adolfo Zambelli", Università di Salerno, Fisciano, Salerno I-84084, Italy.
[i]Department of Chemistry, Energetic Materials Research, Ludwig-Maximilian University, Münich D-81377, Germany.
[j]Department of Chemistry and Department of Earth and Environmental Sciences, University of Illinois Chicago, Chicago, Illinois 60607, United States.

Index

1.Experimental part……………………………………………………………p. 2
2. Synthesis and chemical characterization……………………………p. 2
3. Acid-base equilibria, UV-VIS spectra, and UV-VIS titrations……………p. 8
4. X-ray analysis………………………………………………………………p. 11
5. Hirshfeld surface analysis ……………………………………………p. 19
6. Computational analysis of tautomers…………………………………p. 24
7. Energetic performances of 6……………………………………………p. 27
8. High pressure computations……………………………………………p. 28
9. References………………………………………………………………p. 38

1.Experimental Part

General. All reagents were analytical grade and were used without further purification. Differential thermal analysis (DTA) was performed in air, using a OZM Research DTA 552-Ex instrument, at a heating rate of 5 °C/min. Thermal gravimetric analysis (TGA) was performed under nitrogen flow (20 mL/min) using a PerkinElmer TGA4000 apparatus at a heating rate of 5 °C min. NMR spectra were recorded with Bruker spectrometer operating at 500 MHz, in $CDCl_3$ or [d6]DMSO.

Caution! The compounds in this work are potentially energetic materials that could explode under certain conditions (such as impact, friction, or electric discharge). Experiments should be performed on a small scale. Appropriate safety precautions, including the use of safety shields and personal protections (safety glasses, ear plugs, and gloves), are suggested all the time when handling these compounds.

2. Synthesis and chemical characterization

The synthesis of TTT1 was performed in three steps. In the first step, commercial 5-Amino-1H-1,2,4-triazole-3-carboxylic acid was converted into diamino-triazole T2 by reaction with diaminoguanidine monohydrochloride in polyphosphoric acid (Scheme 1). In the second step, reaction of T2 with acetic anhydride allowed ring-closure and formation of the fused-ring system as a mixture of triamide and tetramide, Scheme 2. In the third step, Scheme 3, basic hydrolysis (and acid recovery) removed the acetyl groups leading to the final product TTT1.

2.1 Synthesis of 1*H*,4'*H* – [3,3' – bis (1, 2, 4-triazolo)]-4',5,5'-triamine (T2)

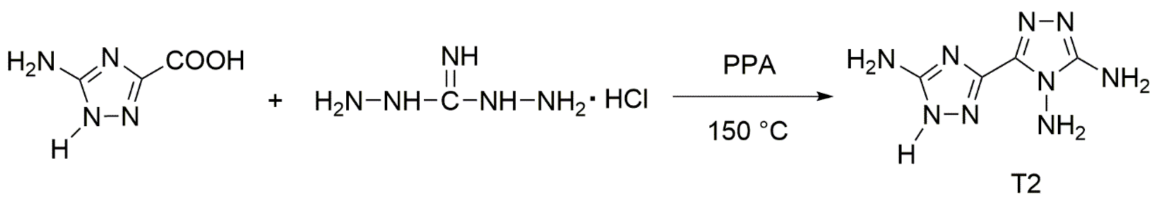

Scheme S1. Synthesis of T2.

The synthesis of T2 was performed according to ref. 1, in which T2 is named compound **1**.

2.2 N-(1-acetyl-3-(1-acetyl-6-methyl-1H-[1,2,4]triazolo[4,3-b][1,2,4]triazol-3-yl)-1H-1,2,4-triazol-5-il)acetammide (Ac3)

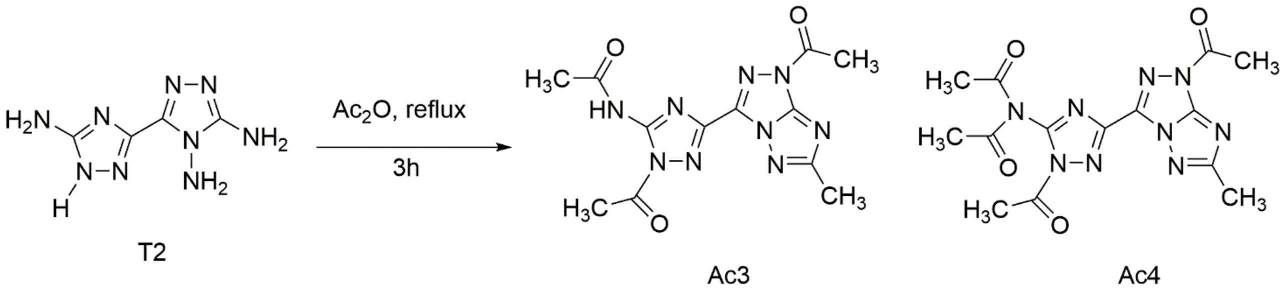

Scheme S2. Synthesis of Ac3 and Ac4.

T2 (4.020 g, 2.22 ·10^{-2} mol) was put into a round bottom flask equipped with a condenser. Then, acetic anhydride (100 mL) was added to the flask. The suspension, under magnetic stirring, was heated to reflux. After few minutes, the refluxing suspension became a yellow-brown solution that was kept at reflux for 3 hours. Then, the flask was cooled to room temperature, and the solution was poured into a beaker. The beaker was put onto a heating plate and, under stirring the solution was kept boiling till its volume reduced to about 20 mL. Then, the solution was cooled to room temperature and poured into toluene (20 mL). A white precipitate of Ac3 was observed, that was recovered by filtration and washed on the filter with hexane and acetone. 2.204 g (6.66 ·10^{-3} mol) of Ac3 were obtained (yield 30%). To the mother liquor after the filtration, 100 mL hexane were added, and a new solid precipitate of Ac4 formed. The precipitate was recovered by filtration, washed with acetone and dried in oven at 100 °C. 1.656 g (4.44 ·10^{-3} mol) of Ac4, were recovered (yield 20%). The total yield was 50%.

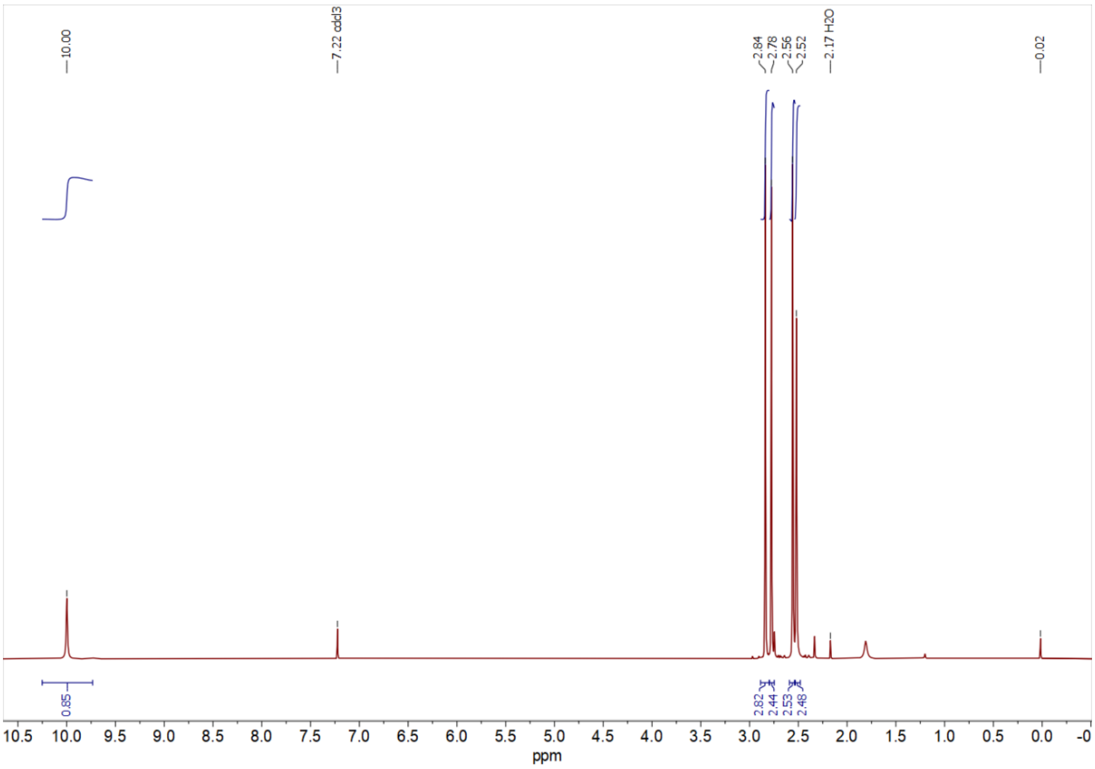

Figure S1. ^{1}H-NMR spectrum of Ac3 in $CDCl_3$. The signal at 7.22 ppm is due to residual non deuterated solvent.

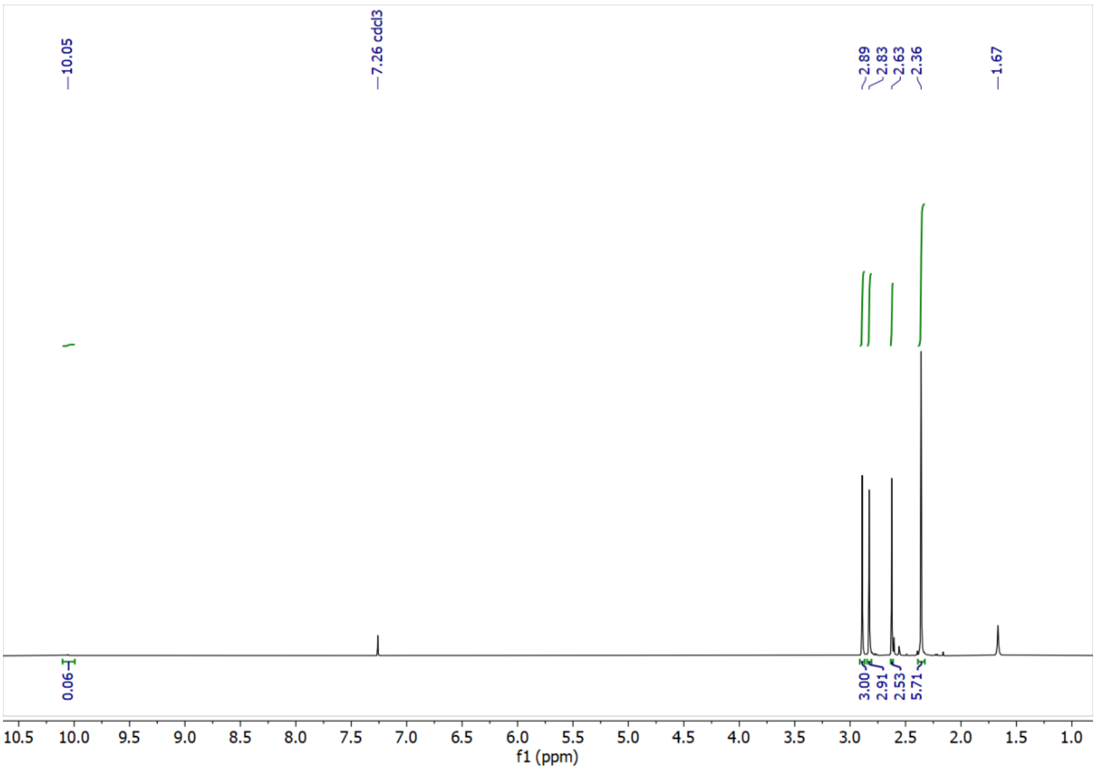

Figure S2. ^{1}H-NMR spectrum of Ac4 in $CDCl_3$. The signal at 7.26 ppm is due to residual non deuterated solvent.

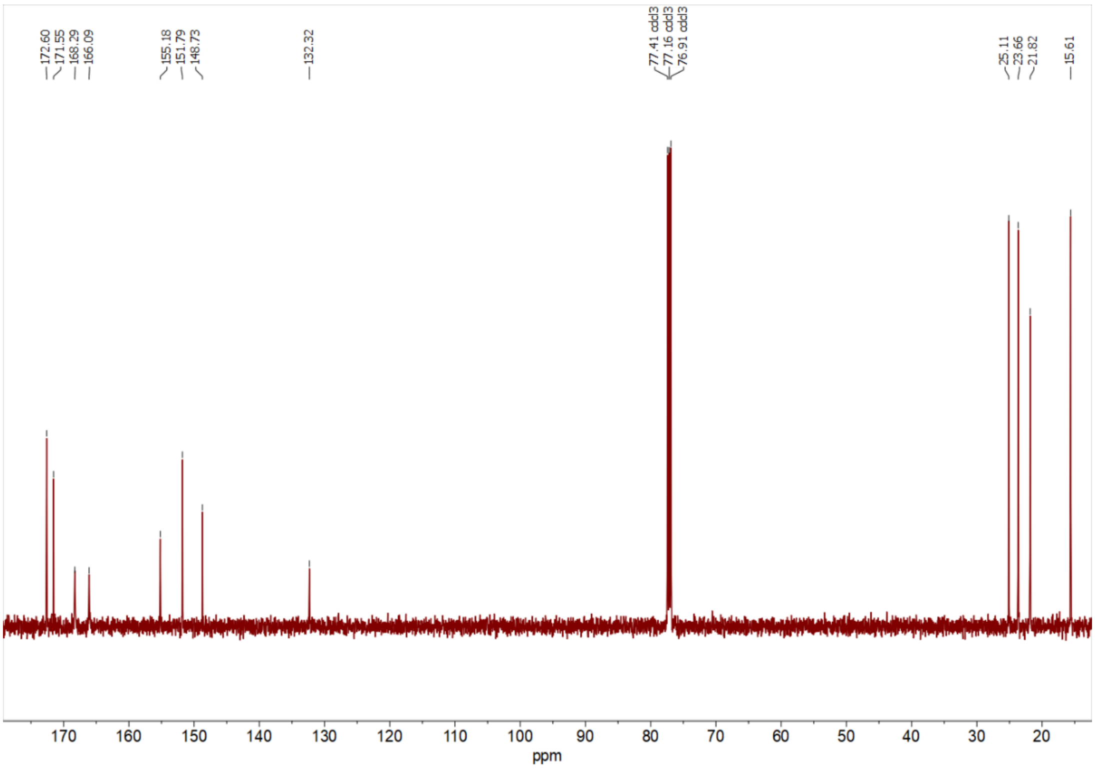

Figure S3. ^{13}C-NMR spectrum of Ac3 in $CDCl_3$. The signals near 80 ppm are due to the solvent.

2.3 Synthesis of 3-{6-methyl-1H-[1,2,4]triazolo[3,2-c][1,2,4]triazole-3-yl}-1H-1,2,4-triazol-5-amino (TTT1)

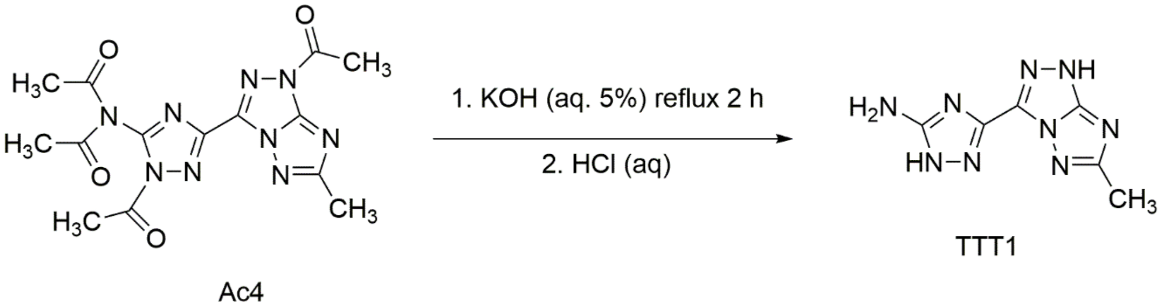

Scheme S3. Synthesis of TTT1 from Ac4.

Ac4 (1.020 g, $2.73{\cdot}10^{-3}$ mol) was transferred into a round bottom flask containing 60 mL of a water solution of KOH at 5% by weight. The flask was connected to a condenser and heated at reflux. After few minutes, the suspension became a pale-yellow solution. The solution was kept at reflux, under stirring for 2 hours. Then, it was cooled to room temperature and poured into a beaker. Under stirring, a solution of HCl 1 M was added in the beaker in small portions, till the pH was 5-6. A white waxy precipitate formed. The suspension was heated at 100 °C under stirring for 1 h. Then, upon cooling to room temperature, the white precipitate went on the bottom of the beaker and was easily filtered, washed with water on the filter and dried in oven at 100 °C. The yield was 72 %. The same procedure, basically with the same yield can be used for the synthesis of TTT1 from Ac3.

Warning: using a more concentrated solution of KOH results in very low yield because of degradation of TTT1 with formation of T2.

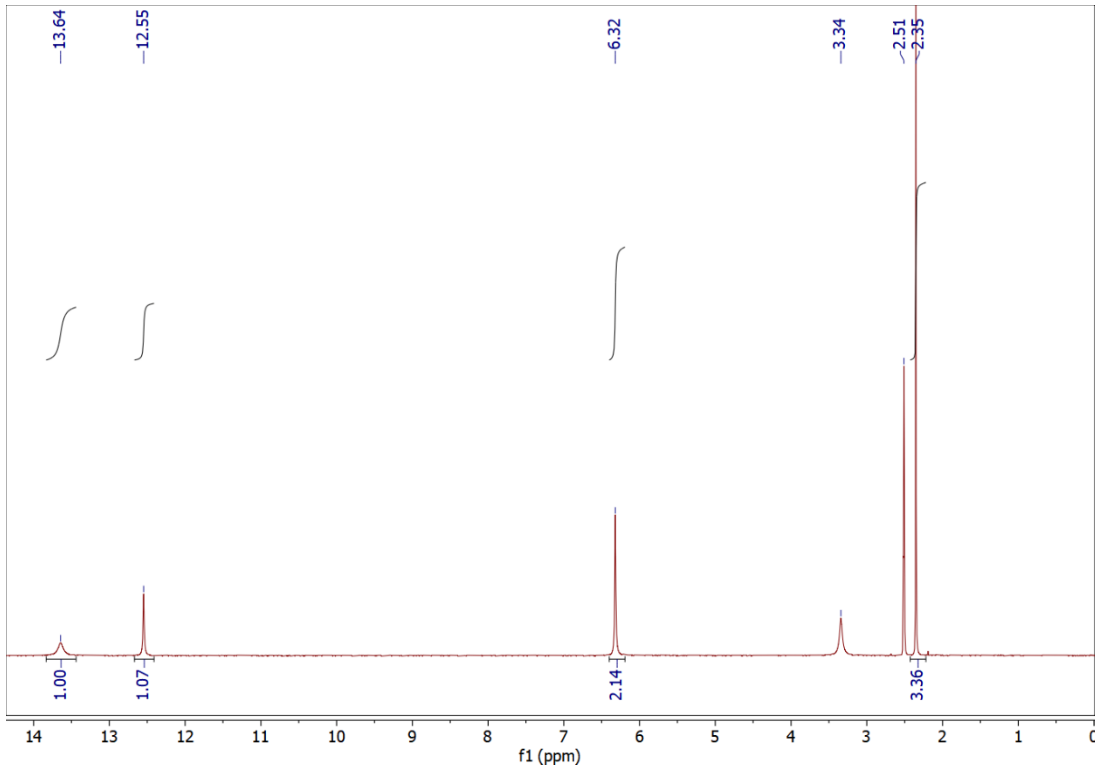

Figure S4. ^{1}H-NMR spectrum of TTT1 in d6-DMSO. The signal at 2.51 ppm is due to residual non deuterated solvent; the signal at 3.34 ppm is due to water.

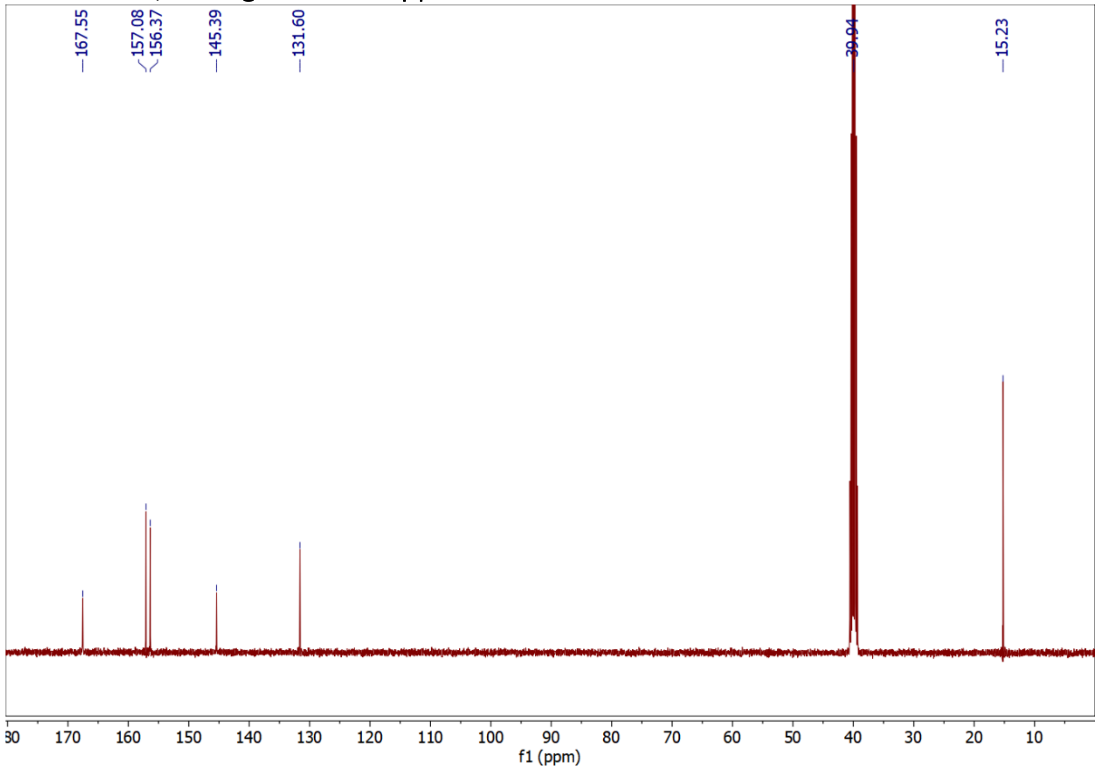

Figure S5. ^{13}C-NMR spectrum of TTT1 in d6-DMSO. The signals near 40 ppm are due to the solvent.

2.4 Synthesis of cationic and anionic salts of TTT1

Cationic salts of TTT1 were obtained by suspending solid TTT1 in a solution of the corresponding mineral acid (HCl, HBr, $HClO_4$, H_2SO_4). The suspension was gently heated until the solid completely dissolved. Then, the solution was concentrated by gently boiling until incipient precipitation. By slowly cooling the hot solution down to room temperature (about 6-7 hours), well developed single crystals of the salts, suitable for X-ray analysis were obtained. The solutions of mineral acids were: HCl 0.01 M for **2**, HCl/HBr 0.1 M for **3** and **5**, HCl 2 M for **4**, H_2SO_4 2 M for **7**. For the anionic salt **8**, TTT1 was suspended in water, and a solution of KOH 1 M was added drop by drop until the solid completely dissolved. The solution was heated at ebullition and concentrated. By slowly cooling to room temperature, single crystals of the potassium salt **8** were obtained.

2.5 Synthesis of energetic perchlorate salt 6

TTT1 (0.652 g, 3.18 mmol) was suspended in 30 mL $HClO_4$ 2 M in a beaker. The suspension was gently heated at ebullition under magnetic stirring till it became a clear solution. Then, the solution was allowed to cool to room temperature. A white microcrystalline precipitate of perchlorate salt **6** formed which was recovered by filtration and washed on the filter with cold water. Yield 0.840 g (2.75 mmol), 86.5%.

The powder diffraction pattern of **6** as obtained by this procedure is shown in Fig. S6. The experimental powder pattern well matches the simulated powder pattern obtained by the single crystal X-ray structure of **6**.

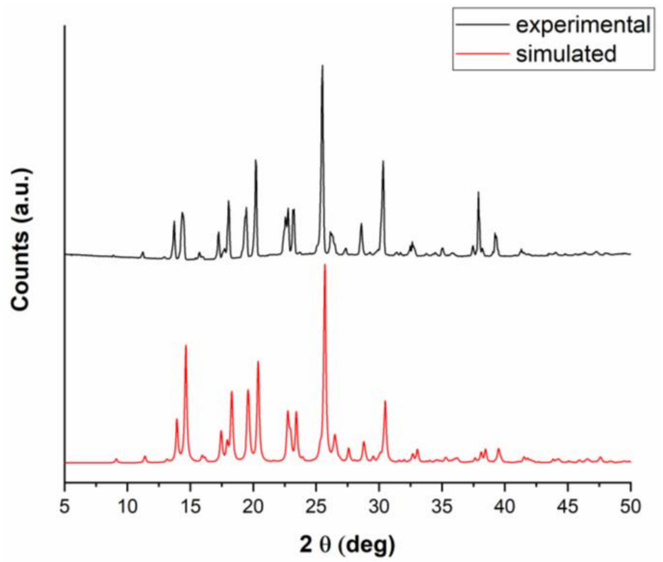

Figure S6. X-ray powder diffraction pattern of **6**, and the simulated powder diffraction pattern of **6** calculated from the single crystal X-ray structure. Radiation used was CuKα.

3.Acid-base equilibria, UV-VIS spectra, and UV-VIS titrations

In this section, TTT1 will also be indicated as H_2L, its singly protonated form as H_3L^+, the doubly protonated form as H_4L^{++}, and the deprotonated form as HL^-. The protolytic equilibria of TTT1 were studied by UV-VIS absorption spectroscopy in 0.5 M NaCl, as the ionic medium. The TTT1 stock solutions were prepared starting from the solid. Deionized and doubly distilled water was used to prepare all aqueous solutions. NaCl (Fluka, dried overnight at 120 °C) was used to prepare the ionic medium solutions. Stock solutions of HCl (Carlo Erba p.a.) were standardized against $KHCO_3$ using methyl red as indicator, with a reproducibility of 0.1 %. NaOH (Baker p.a.) stock solutions were standardized against standardized HCl solutions. The experiments were performed as acid-base titrations at constant total concentration of TTT1 (C= 70 μM). The investigated pH spans between 0.3 and 12. For each experimental point at 2.5<pH<10, the equilibrium free proton concentration was evaluated from the measured electromotive force at the ends of the galvanic cell GE/TS/RE, where TS indicates the Test solution, GE is the glass electrode and RE is a reference electrode (0.5 M NaCl|Hg_2Cl_2|Hg(Pt)) placed outside but electrically connected to TS through a salt bridge. The Nernst potential of the cell, E(mV), can be written as $E= E_0 + 59.16 \log [H^+] + E_j$ where E_j= liquid junction potential due to the replacement of Na^+ with H^+.[2] The evaluation of the constant of the glass electrode, E_0, was performed in the first stage of each experiment by a coulometric titration, using the Gran method.[3] The test solutions at 0.3<pH<2 were obtained analytically. All the experiments were carried out in air in a thermostat, at 25.00 ± 0.03 °C. Potentiometric experimental data were collected by means of an automatic data acquisition system based on Hewlett-Packard (HP) instrumentation. The glass membrane electrodes reversible to protons were supplied by Metrohm. Highly precise (± 0.02 mV) emf measurements were made by adapting the impedance of the glass electrode through operational amplifiers. Coulometric variations of the solution composition were carried out using a Hewlett Packard "DC Power Supply". The intensity of the current in the electrolysis circuit was measured from the potential drop at the ends of a calibrated resistance; the current density was set at about 1 mA/cm^2. Absorption spectra, Fig. S7, were recorded with a Varian Cary 50 UV-Vis spectrophotometer using 1 cm cell. The primary spectrophotometric data (Absorbance, pH, λ) were elaborated both graphically[4] and numerically, by the HYPERQUAD program.[5] In Figure S8, the continuous curves were obtained by fitting the experimental points with an equation that relates the Absorbance of the solution (at a given wavelength) with the pH, the analytical concentration of TTT1, and the equilibrium constants, by

assuming the law of additivity of absorbances (Bouger-Lambert-Beer equation). The Bouger-Lambert-Beer equation is now explicitly derived for the system TTT1 (H_2L).

From the Lambert-Beer law, assuming additivity of the absorbances, it is

$$A^{\lambda} = b\left(\sum_i \varepsilon_i^{\lambda} c_i\right) = b\big(\varepsilon_1^{\lambda}[H_4L^{++}] + \varepsilon_2^{\lambda}[H_3L^{+}] + \varepsilon_3^{\lambda}[H_2L] + \varepsilon_4^{\lambda}[HL^{-}]\big) \quad (1)$$

in which *b* is the optical path of the cell (1 cm) and ε_i^{λ} is the molar extinction coefficient of the species *i* at the given wavelength. The analytical concentration of TTT1 (H_2L), *C*, is given by

$$C = [H_4L^{++}] + [H_3L^{+}] + [H_2L] + [HL^{-}] \quad (2)$$

The acid-base conditional equilibrium constants are

$$K_{a1} = \frac{[H_3L^{+}]\cdot[H_3O^{+}]}{[H_4L^{++}]}, K_{a2} = \frac{[H_2L]\cdot[H_3O^{+}]}{[H_3L^{+}]}, K_{a3} = \frac{[HL^{-}]\cdot[H_3O^{+}]}{[H_2L]} \quad (3)$$

From these, it is possible to express the concentration of all species but one (e. g. the neutral one), as a function of the equilibrium constants and the concentration of H_3O^+:

$$[H_4L^{++}] = \frac{[H_3L^{+}]\cdot[H_3O^{+}]}{K_{a1}}, [H_3L^{+}] = \frac{[H_2L]\cdot[H_3O^{+}]}{K_{a2}}, [HL^{-}] = \frac{[H_2L]\cdot K_{a3}}{[H_3O^{+}]}$$

that is

$$[H_4L^{++}] = \frac{[H_2L]\cdot[H_3O^{+}]^2}{K_{a1}\cdot K_{a2}}, [H_3L^{+}] = \frac{[H_2L]\cdot[H_3O^{+}]}{K_{a2}}, [HL^{-}] = \frac{[H_2L]\cdot K_{a3}}{[H_3O^{+}]} \quad (4)$$

Putting (4) in (2) it is

$$C = \frac{[H_2L]\cdot[H_3O^{+}]^2}{K_{a1}\cdot K_{a2}} + \frac{[H_2L]\cdot[H_3O^{+}]}{K_{a2}} + [H_2L] + \frac{[H_2L]\cdot K_{a3}}{[H_3O^{+}]} \quad (5)$$

So, it is

$$[H_2L] = \frac{CK_{a1}K_{a2}[H_3O^{+}]}{[H_3O^{+}]^3 + K_{a1}[H_3O^{+}]^2 + K_{a1}K_{a2}[H_3O^{+}] + K_{a1}K_{a2}K_{a3}} \quad (6)$$

Putting (4) and (6) in (1) yields

$$A = b\cdot\left(\varepsilon_1^{\lambda}\frac{[H_2L]\cdot[H_3O^{+}]^2}{K_{a1}K_{a2}} + \varepsilon_2^{\lambda}\frac{[H_2L]\cdot[H_3O^{+}]}{K_{a2}} + \varepsilon_3^{\lambda}[H_2L] + \varepsilon_4^{\lambda}\frac{[H_2L]\cdot K_{a3}}{[H_3O^{+}]}\right) \quad (7)$$

$$A = b\cdot[H_2L]\left(\varepsilon_1^{\lambda}\frac{[H_3O^{+}]^2}{K_{a1}K_{a2}} + \varepsilon_2^{\lambda}\frac{[H_3O^{+}]}{K_{a2}} + \varepsilon_3^{\lambda} + \varepsilon_4^{\lambda}\frac{K_{a3}}{[H_3O^{+}]}\right) \quad (8)$$

$$A = b\cdot\frac{CK_{a1}K_{a2}[H_3O^{+}]}{[H_3O^{+}]^3 + K_{a1}[H_3O^{+}]^2 + K_{a1}K_{a2}[H_3O^{+}] + K_{a1}K_{a2}K_{a3}}\left(\varepsilon_1^{\lambda}\frac{[H_3O^{+}]^2}{K_{a1}K_{a2}} + \varepsilon_2^{\lambda}\frac{[H_3O^{+}]}{K_{a2}} + \varepsilon_3^{\lambda} + \varepsilon_4^{\lambda}\frac{K_{a3}}{[H_3O^{+}]}\right) \quad (9)$$

$$A = b \cdot \frac{C}{[H_3O^+]^3 + K_{a1}[H_3O^+]^2 + K_{a1}K_{a2}[H_3O^+] + K_{a1}K_{a2}K_{a3}} \left(\varepsilon_1^\lambda [H_3O^+]^3 + \varepsilon_2^\lambda K_{a1}[H_3O^+]^2 + \varepsilon_3^\lambda K_{a1}K_{a2}[H_3O^+] + \varepsilon_4^\lambda K_{a1}K_{a2}K_{a3}\right) \quad (10)$$

and, finally, the Bouger-Lambert-Beer equation

$$A = b \cdot \frac{C}{10^{-3pH} + 10^{-2pH-pK_{a1}} + 10^{-pH-pK_{a1}-pK_{a2}} + 10^{-pK_{a1}-pK_{a2}-pK_{a3}}} \left(\varepsilon_1^\lambda 10^{-3pH} + \varepsilon_2^\lambda 10^{-2pH-pK_{a1}} + \varepsilon_3^\lambda 10^{-pH-pK_{a1}-pK_{a2}} + \varepsilon_4^\lambda 10^{-pK_{a1}-pK_{a2}-pK_{a3}}\right)$$

The UV-VIS spectra, at constant total concentration and different pH, are reported in Fig. S7 for TTT1.

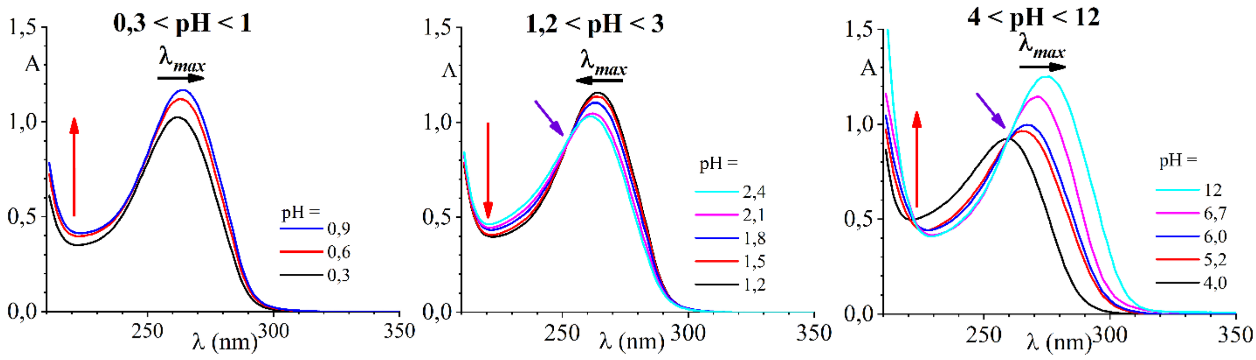

Figure S7. UV−VIS absorption spectra of TTT1 at constant total concentration, C = 70 μM, in NaCl 0.5 M recorded at 0.3 ≤ pH ≤ 12. The spectra have been grouped in three sets of curves for easier lecture.

The plots of Absorbance *vs* pH at fixed wavelength and the fitting of the points using the Bouger-Lambert-Beer equation are reported in Fig. S8.

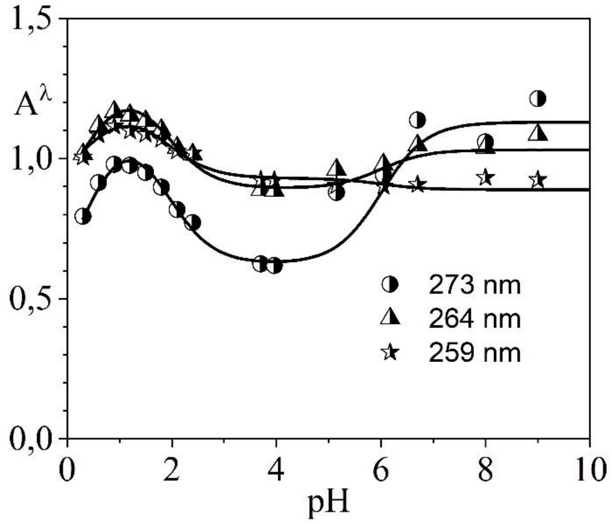

Figure S8. UV-VIS titration curves of TTT1, at three different wavelengths. The continuous curves have been constructed by fitting the experimental points (absorbance *vs* pH data taken from Fig. S7) with the Bouger-Lambert-Beer equation.

4.X-ray analysis

4.1 Data collection and refinement at 0.1 MPa

All data for crystal structure determinations were measured on a Bruker-Nonius Kappa CCD diffractometer equipped with Oxford Cryostream 700 apparatus, using graphite monochromated MoKα radiation (λ = 0.71073 Å). Reduction of data and semiempirical absorption correction were done using the SADABS program.[6] The structures were solved by direct methods (SIR97 program[7]) and refined by the full-matrix least-squares method on F^2 using the SHELXL-2016 program[8] with the aid of the program WinGX.[9] H atoms bonded to N, that are essential in the identification of tautomers, and those bonded to O in water molecules, were clearly found in difference Fourier maps as the first maxima, and in some cases their coordinates were refined. For all H atoms, U_{iso} =1.2 × U_{eq} of the carrier atom was assumed. Full crystal and refinement data are summarized in Tables S1 and S2. The analysis of the crystal packing was performed using the program Mercury.[10] CCDC deposition numbers from 2475986 to 2475990 and from 2475992 to 2475994 contain the supplementary crystallographic data for this article.

Table S1. Crystal, collection, and refinement data for the structures described in the paper.

	1	2	3	4
Chemical formula	$C_6H_7N_9{\cdot}H_2O$	$C_6H_8N_9Cl{\cdot}C_6H_7N_9{\cdot}2H_2O$	$C_6H_8N_9Cl$	$(C_6H_9N_9)_2Cl_4{\cdot}3H_2O$
M_r	223.22	482.90	241.66	610.29
System	Monoclinic	Monoclinic	Orthorhombic	Triclinic
Space group	$P2_1/c$	$P2_1$	$Pnma$	$P\bar{1}$
Temp. (K)	200	293	150	293
a (Å)	7.2403(7)	7.398(3)	12.1163(9)	6.7330(8)
b (Å)	9.9644(9)	12.092(5)	6.4333(5)	11.309(2)
c (Å)	13.1965(13)	11.398(5)	12.2978(9)	17.065(4)
α (°)	90	90	90	101.069(12)
β (°)	95.307(3)	98.59(2)	90	90.812(13)
γ (°)	90	90	90	99.169(12)
V (Å^3)	947.98(16)	1008.2(7)	958.59(12)	1257.6(4)
Z	4	2	4	2
λ	Mo Kα	Mo Kα	Mo Kα	Mo Kα
μ (mm^{-1})	0.120	0.247	0.387	0.527
Cryst. size (mm)	0.03× 0.02 × 0.02	0.40 × 0.30 × 0.10	0.08 × 0.04 × 0.02	0.40 × 0.30 × 0.20

Meas., ind. reflns	21662, 2304	10087, 4106	22413, 1298	9479, 5336
R_{int}	0.041	0.033	0.0460	0.0425
θ_{max} (°)	28.561	27.494	28.279	27.498
$R[I > 2\sigma(I)]$, wR(all), S	0.046, 0.1421, 1.08	0.0391, 0.0971, 1.07	0.0343, 0.0980, 1.07	0.0421, 0.0992, 1.10
Data	2304	4106	1298	5336
Parameters	164	333	112	390
Restraints	0	5	0	1
$\Delta\rho_{max}$, $\Delta\rho_{min}$ (e Å^{-3})	0.392, −0.335	0.188, −0.208	0.619, -0.237	0.238, -0.272
CCDC	2475992	2475993	2475988	2475989

Table S2. Crystal, collection, and refinement data for the structures described in the paper.

	**5**	**6**	**7**	**8**
Chemical formula	$C_6H_8N_9Br{\cdot}H_2O$	$C_6H_8N_9ClO_4$	$(C_6H_9N_9)SO_4{\cdot}3H_2O$	$K(C_6H_6N_9){\cdot}3H_2O$
M_r	304.14	305.66	357.33	297.35
System	Orthorhombic	Monoclinic	Monoclinic	Orthorhombic
Space group	$Pbcn$	$P2_1/c$	$P2_1/c$	$Pna2_1$
Temp. (K)	173	173	173	173
a (Å)	20.9100(14)	11.327(4)	7.479(3)	22.805(6)
b (Å)	13.762(4)	7.777(3)	12.433(5)	3.778(2)
c (Å)	7.564(6)	15.640(4)	15.737(5)	13.857(4)
α (°)	90	90	90	90
β (°)	90	121.16(2)	96.34(2)	90
γ (°)	90	90	90	90
V (Å^3)	2176.6(18)	1178.9(7)	1454.4(9)	1193.9(8)
Z	8	4	4	4
λ	Mo $K\alpha$	Mo $K\alpha$	Mo $K\alpha$	Mo $K\alpha$
μ (mm^{-1})	3.780	0.359	0.279	0.469
Cryst. size (mm)	0.3 × 0.1 × 0.1	0.4 × 0.3 × 0.3	0.4 × 0.2 × 0.2	0.4 × 0.2 × 0.1
Meas., ind. reflns	11695, 2476	11197, 2689	17996, 3311	4550, 2278
R_{int}	0.0501	0.0246	0.0451	0.0632
θ_{max} (°)	27.517	27.533	27.505	27.489

R[I > 2σ(I)], *wR*(all), *S*	0.0386, 0.0805, 1.07	0.0346, 0.0957, 1.04	0.0351, 0.0938, 1.07	0.0568, 0.1479, 1.12
Data	2476	2689	3311	2278
Parameters	176	197	245	200
Restraints	1	0	1	13
$\Delta\rho_{max}$, $\Delta\rho_{min}$ (e Å^{-3})	0.704, -0.513	0.513, -0.420	0.347, -0.382	0.477, -0.645
CCDC	2475987	2475994	2475990	2475986

4.2 High pressure X-ray diffraction analysis of 6

A single crystal of the energetic perchlorate salt **6** was mounted in a four-post diamond anvil cell (DAC) with diamonds having culet diameters of 500 µm and a rhenium gasket was prepared by pre-indenting it to 20 GPa, and laser drilling the hole to 250 µm diameter and Parabar 10312 (previously known as Paratone) was used as the pressure-transmitting medium. The pressure in the DAC chamber was determined by ruby fluorescence before and after each diffraction measurement.[11] The single crystal data were collected by a Rigaku Oxford Diffraction Synergy-S diffractometer equipped with a HyPix6000HE detector and operating with MoKα radiation. The data collection routine, unit cell refinement, and data processing were carried out using the CrysAlisPro program.[12] Unit cell parameters at different pressures are reported in Table S3.

Table S3. Experimental values of unit cell parameters of **6** at different pressures.

	0.1 MPa	0.4 GPa	0.8 GPa	1.2 GPa	2.1 GPa	2.8 GPa
Sp. Group	$P2_1/c$					
a (Å)	11.406(4)	11.29(8)	11.24(3)	11.15(9)	10.48(7)	10.15(8)
b (Å)	7.741(8)	7.6902(8)	7.6893(6)	7.6388(18)	7.7789(14)	7.815(2)
c (Å)	15.841(4)	15.46(12)	15.37(4)	15.16(13)	14.19(11)	14.06(14)
β (°)	121.74(3)	121.1(12)	120.8(4)	120.5(14)	115.05(12)	112.76(15)
V (Å^3)	1189.84	1148(29)	1142(7)	1113(24)	1049(18)	1028(23)
Z/Z’	4/1	4/1	4/1	4/1	4/1	4/1
D_x (g/cm^3)	1.707	1.767	1.777	1.824	1.935	1.975

4.3 Description of the packings

In the packing of **1**, Fig. S9, molecules of TTT1 are associated in H-bonded chains that run parallel to b-axis, being wrapped around the 2_1 screw axes of the $P2_1/c$ space group. The chains are laterally H-bonded through the water molecules.

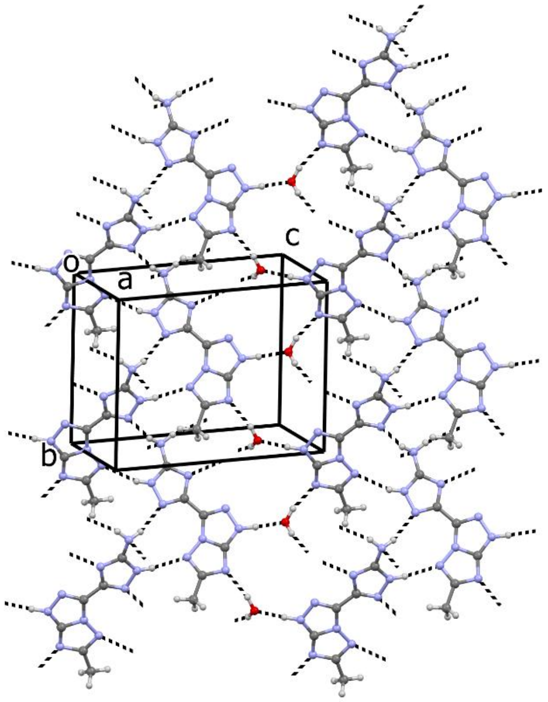

Figure S9. Partial crystal packing of **1**.

The crystal packing of **2** is of layered nature, Fig. S10. Planar layers are formed by neutral and cationic molecules placed in regular alternate sequence. Within each layer, molecules are bonded by ionic and H-bonds. The layers are parallel to lattice planes with Miller indices 201, and this is consistent with 201 being the most intense reflection of the whole diffraction pattern. The spacing of the planes is $d = 3.30$ Å.

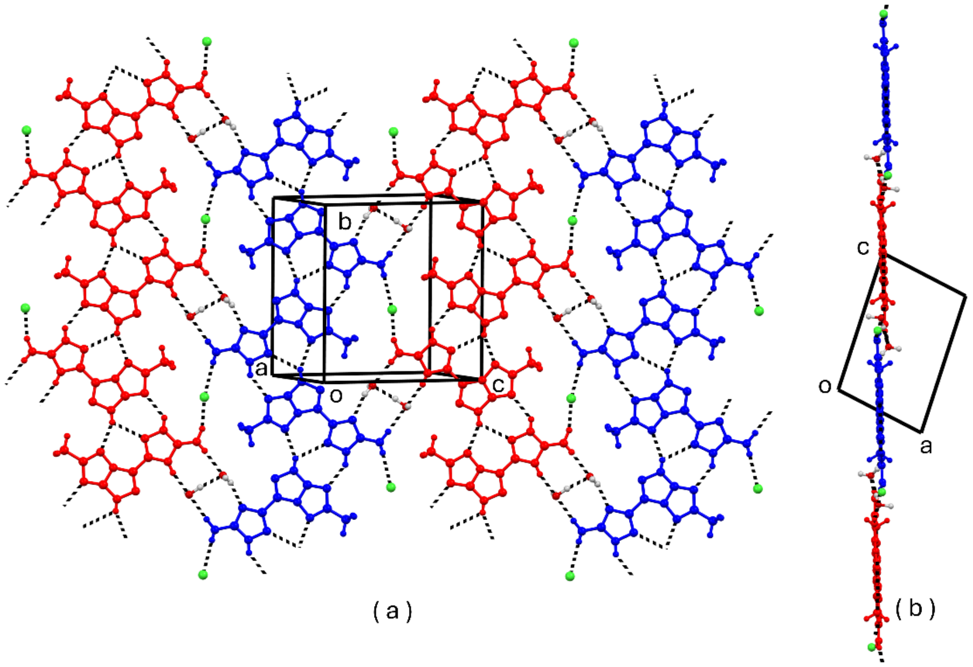

Figure S10. Crystal packing of **2**. Neutral molecules are shown in blue, molecules of the monocation are shown in red. Chloride anions are in green. (a) Face view of a layer; (b) edge view (along b-axis) of the same layer.

The crystal packing of **3** is also of layered nature, Fig. S11. Planar layers parallel to (a, c) and formed by molecules bonded by ionic and H-bonds are stacked along b-axis. The layers are parallel to lattice planes with Miller indices 020, and this is consistent with 020 being the most intense reflection of the whole diffraction pattern. The spacing of the planes is $d = 3.22$ Å.

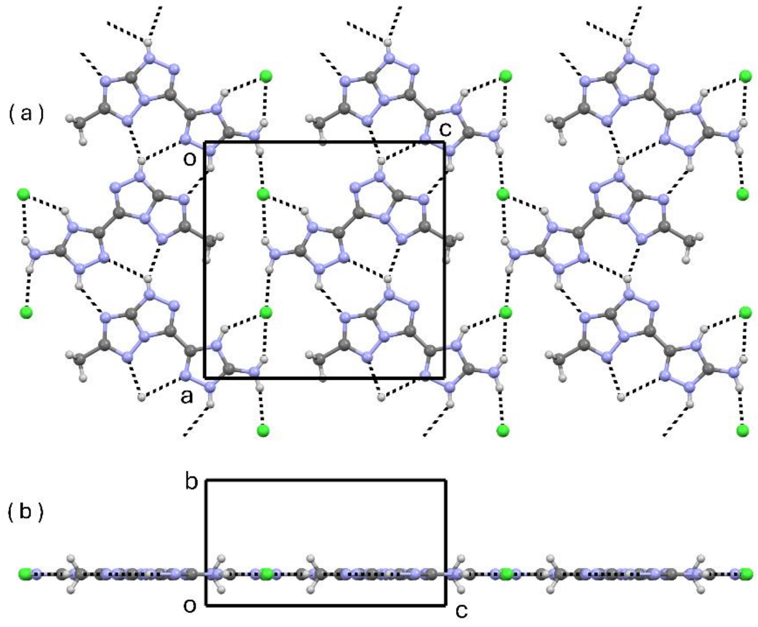

Figure S11. Crystal packing of **3**. (a) view down b-axis; (b) view down a-axis.

The crystal packing of **4** is also of layered nature, Fig. S12. Planar layers parallel to (b, c) and formed by molecules bonded by ionic and H-bonds are stacked along a-axis. The layers are parallel to lattice planes with Miller indices 020, and this is consistent with 020 being the most intense reflection of the whole diffraction pattern. The spacing of the planes is $d = 3.32$ Å.

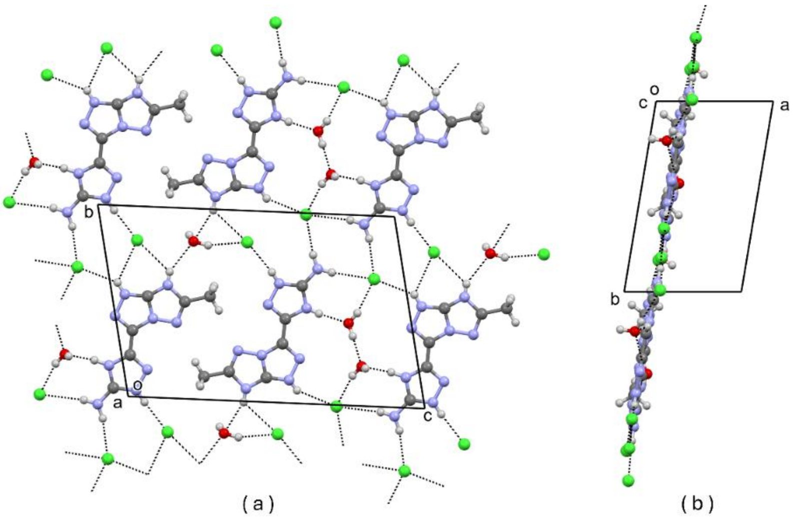

Figure S12. Crystal packing of **4**. (a) a layer projected down a; (b) the same layer viewed down c.

In the packing of **5**, Fig. S13, ribbons of H-bonded molecules are present. The ribbons, parallel to b axis, are stacked parallel to each other in the c-axis direction and in zig-zag fashion in the a-axis direction. The water molecule is saturated for H-bonding: it is acceptor from two N-H and donor to two Br^- ions. This accounts for the long-time stability of crystals of **5**, that are stable at room temperature in the air for several weeks without loss of water.

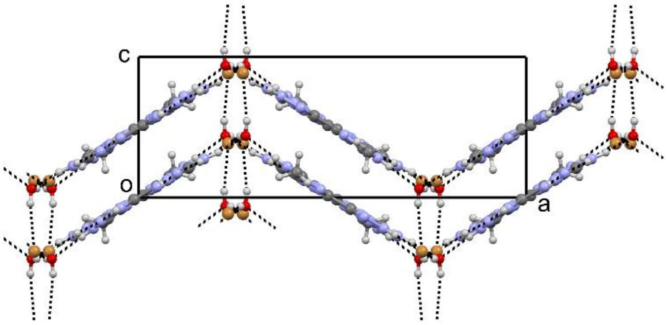

Figure S13. Packing of **5** viewed down b.

The crystal packing of **7** is also of layered nature, Fig. S14. Planar layers are formed by cationic molecules, water molecules and sulfate anions bonded to each other by ionic and H-bonds. The layers are parallel to lattice planes with Miller indices 202, and this is consistent with 202 being the most intense reflection of the whole diffraction pattern. The spacing of the planes is $d = 3.22$ Å.

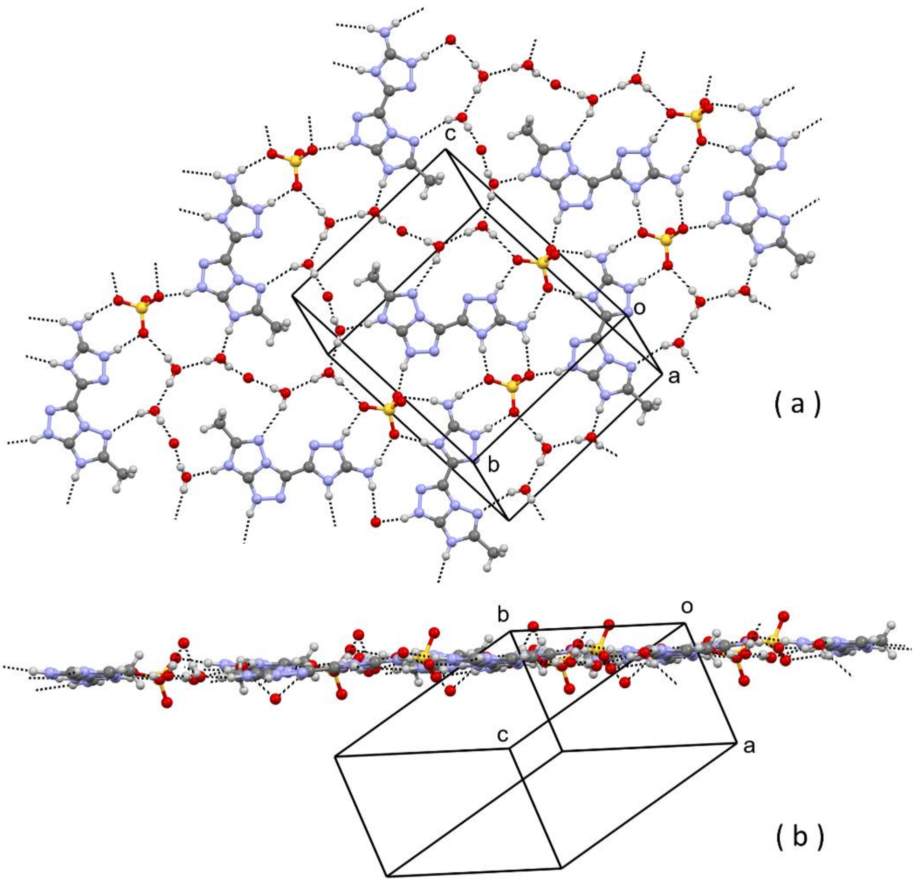

Figure S14. Packing of **7**. (a) Face view of a layer; (b) edge view of the same layer.

The crystal packing of **8** is shown in Fig. S15. The potassium salt crystallizes as trihydrate. One water molecule is coordinated to K^+, the other two are not coordinated. The two non-coordinated water molecules are saturated for what concerns H-bonds, and in fact the crystals are stable in air for weeks. The H^+ is lost by the bicycle and not by the single triazole ring, as expected. The coordination around K^+ is driven by steric and electrostatic factors because K^+ has a closed shell electronic configuration. The coordination is due to two water molecules and to N atoms of the triazolo-triazolate anion. The triazolate can act as bidentate chelate ligand in two ways: (N1, N8) with formation of a pentatomic chelate ring and (N4, N6) in which the chelate ring is hexatomic. This latter should be preferred because the resonance formulae delocalize the negative charge of the anion on N4 and N6. At any rate, both chelating modes are present in the crystal structure, Fig. S15(a). Besides the two chelating anions and two water molecules, the coordination sphere of K^+ is completed by three further N atoms, for a total coordination number of 9, Fig. S15(b). Moreover, water molecules and N1 and N6 atoms of the two chelate rings are at bridge between K^+ cations along b-axis. On the other hand, each triazolotriazolate anion acting as chelate in two ways (N1, N8)

and (N4, N6) in opposite sides, produces coordination chains running parallel to c, Fig.S15(a). The result is the formation of infinite layers of cations/anions in the plane (b,c), Fig. S15(b). These layers are stacked along a and are bonded to each other by the non-coordinated water molecules that are involved in H-bonds between each other, or with the coordinated water molecule or with one N atom of the triazolate anion.

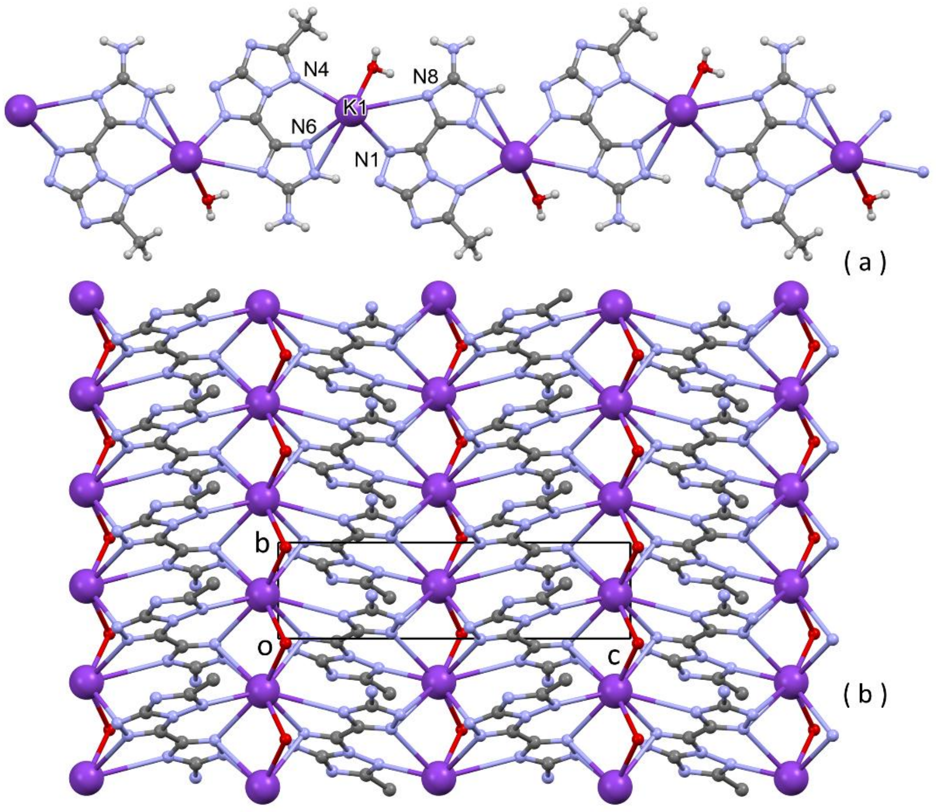

Figure S15. Crystal packing of **9**. (a) a coordinated chain running along c; (b) a coordination layer parallel to (b, c).

5.Hirshfeld surface analysis

2D Hirshfeld fingerprint plots for compounds **1**-**7** were calculated with the program CrystalExplorer v. 25-09[13] and are shown in Figs. S16-S22. For each contact, the fraction (percent) of the contact over the total contacts is also shown. The fingerprint plot is a graphical two-dimensional map that indicates the distribution of the interactions for a single molecule in the crystal. In the plot, for each point of the Hirshfeld surface enveloping the molecule in the crystal, the distance d_i to the nearest atom inside the surface and the distance d_e to the nearest atom outside the surface are reported. The colour of each point in the plot is related to the abundance of that interaction, from blue (low) to green (high) to red (very high).

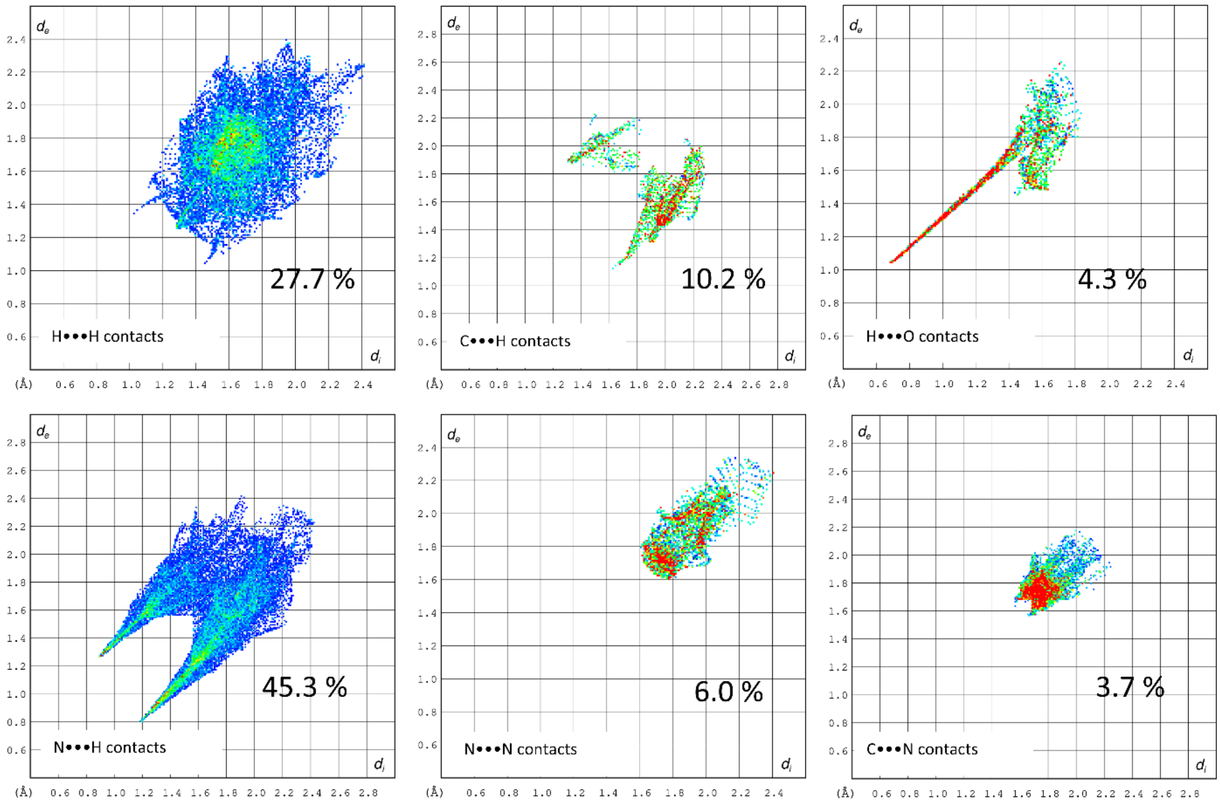

Figure S16. Hirshfeld 2D fingerprint plots for different intermolecular contacts of **1**. The percent of the contact is also indicated.

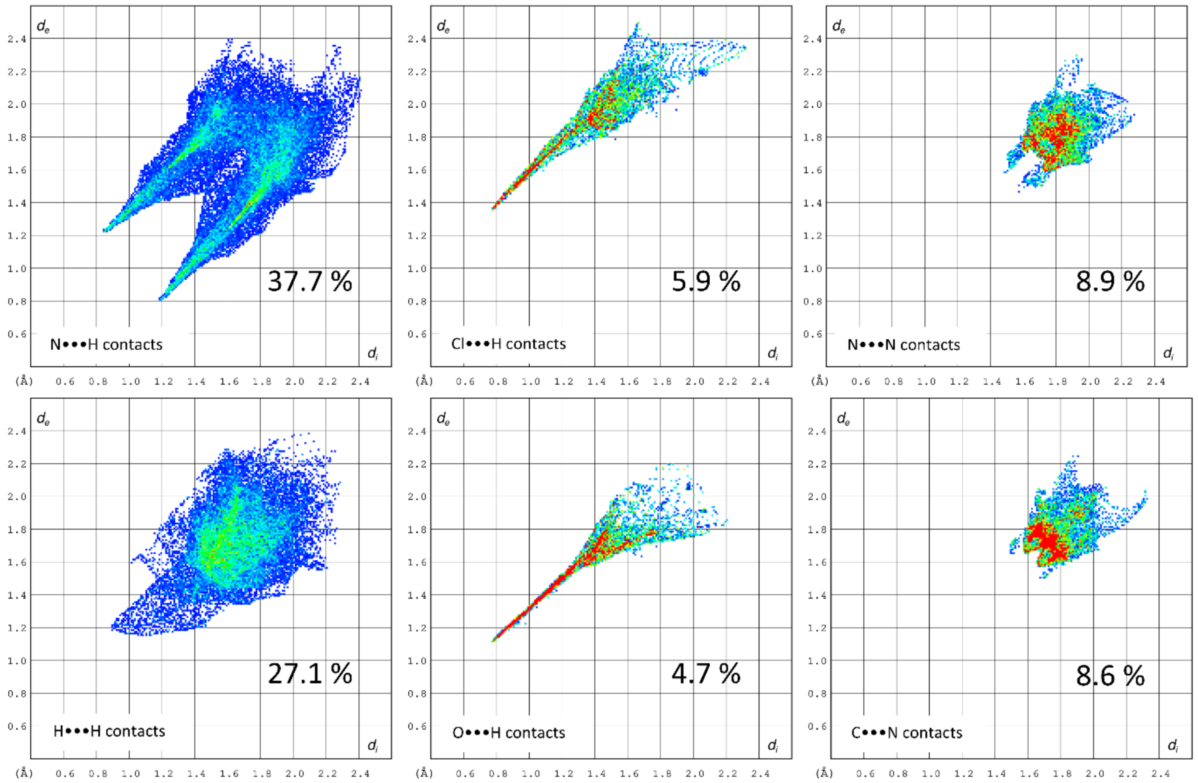

Figure S17. Hirshfeld 2D fingerprint plots for different intermolecular contacts of **2**. The percent of the contact is also indicated.

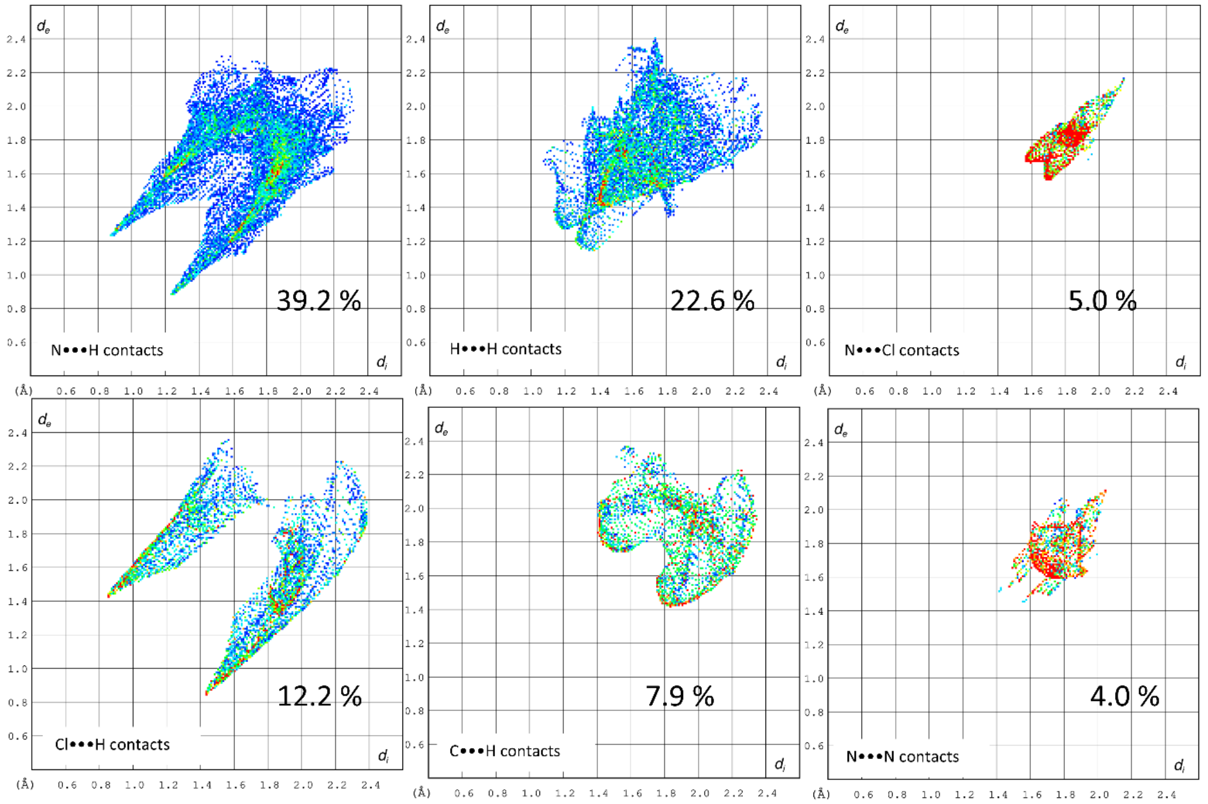

Figure S18. Hirshfeld 2D fingerprint plots for different intermolecular contacts of **3**. The percent of the contact is also indicated.

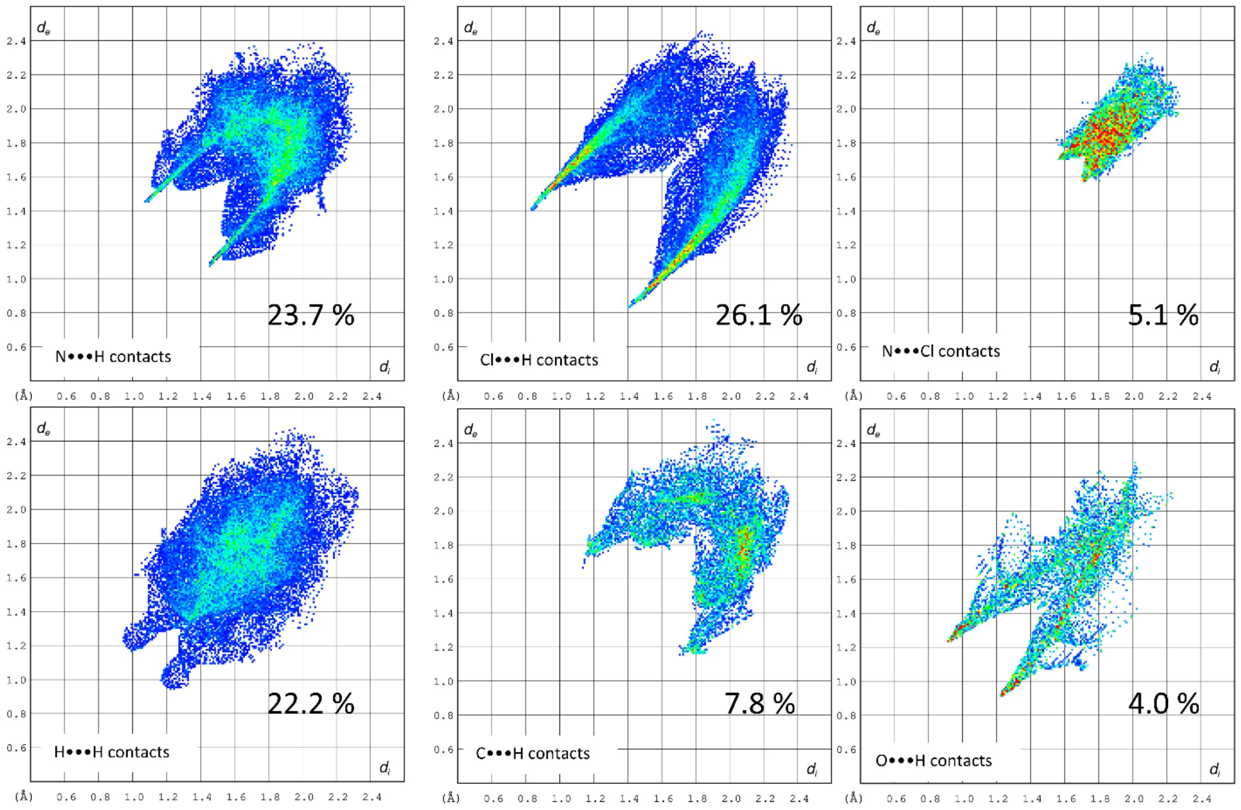

Figure S19. Hirshfeld 2D fingerprint plots for different intermolecular contacts of **4**. The percent of the contact is also indicated.

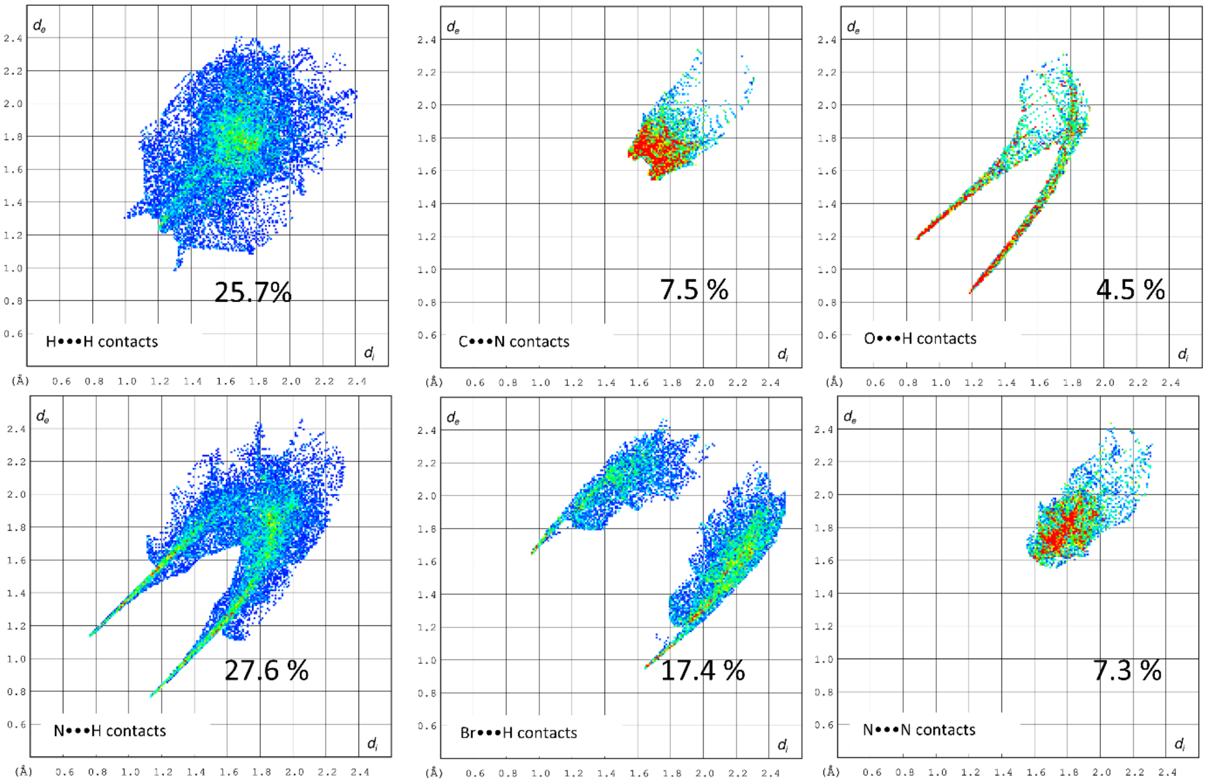

Figure S20. Hirshfeld 2D fingerprint plots for different intermolecular contacts of **5**. The percent of the contact is also indicated.

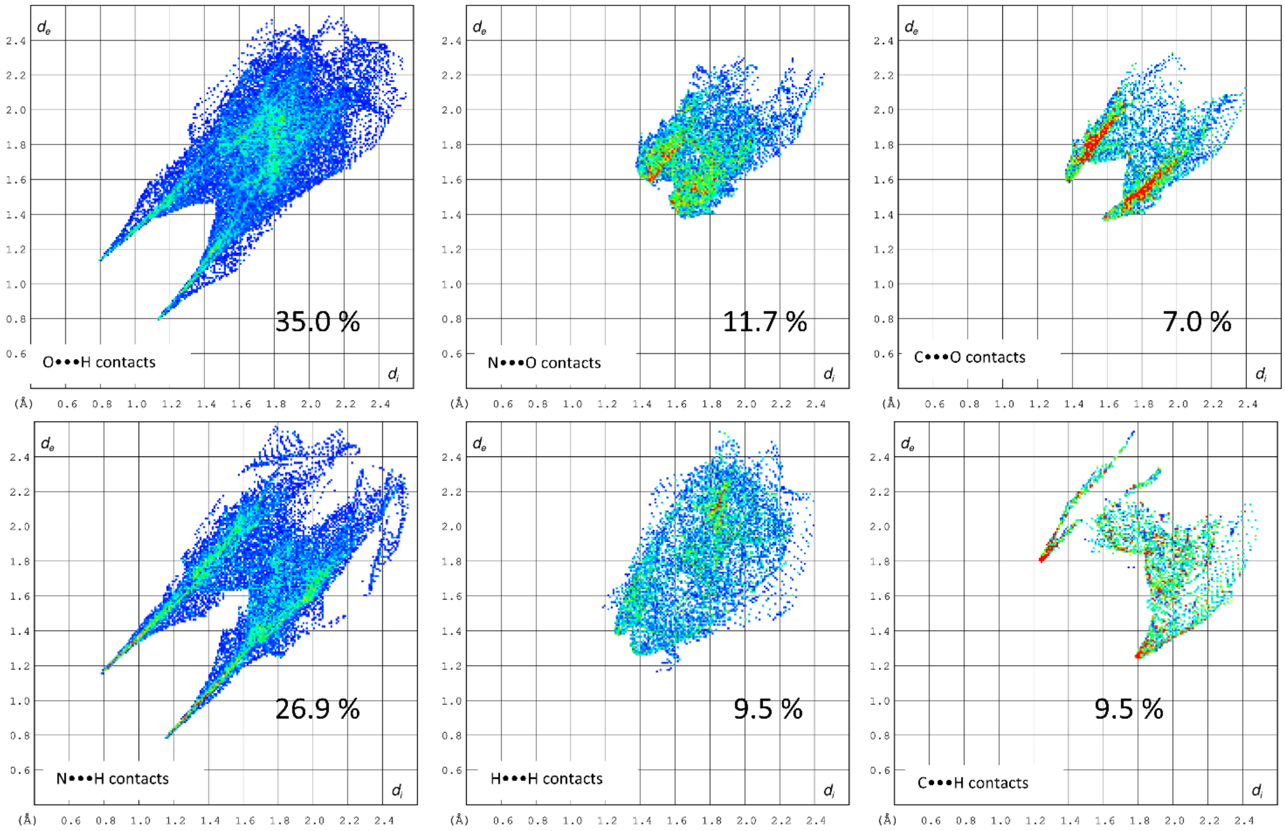

Figure S21. Hirshfeld 2D fingerprint plots for different intermolecular contacts of **6**. The percent of the contact is also indicated.

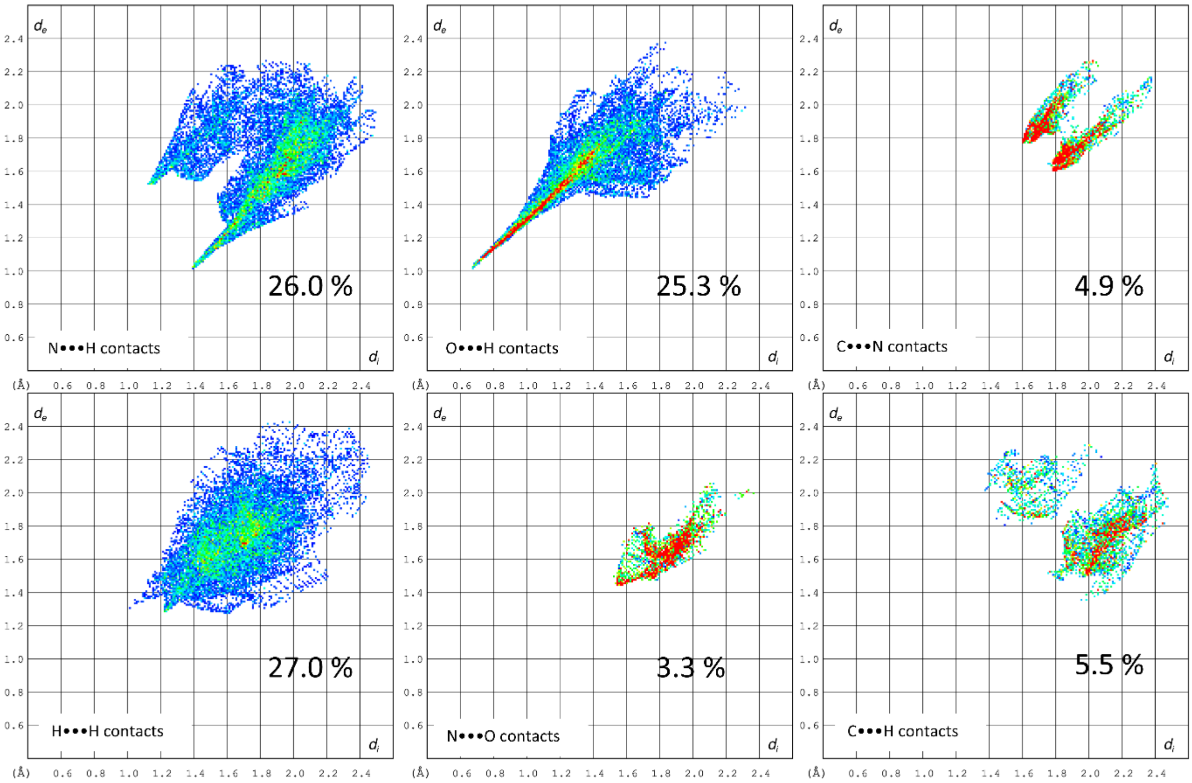

Figure S22. Hirshfeld 2D fingerprint plots for different intermolecular contacts of **7**. The percent of the contact is also indicated.

Among the most abundant contacts, for all the compound, is the N···H one. This includes the strong N-H···N or O-H···N hydrogen bonds and appears, in the 2D plot, as typical spikes symmetrically disposed with respect to the principal diagonal of the plot. Similar spikes also appear for the O···H contact in the structure of **6**, in which O atoms of the perchlorate are H bonding acceptors from N-H donors. The red coloured zone centred at (d_i, d_e) = (1.8, 1.8) and present in many plots, under N···N or C···N contacts, corresponds to the π–π stacking interactions.[14]

The relevance of H-bonding in the packing of compounds is further corroborated by the histogram of contacts reported in Fig. S23.

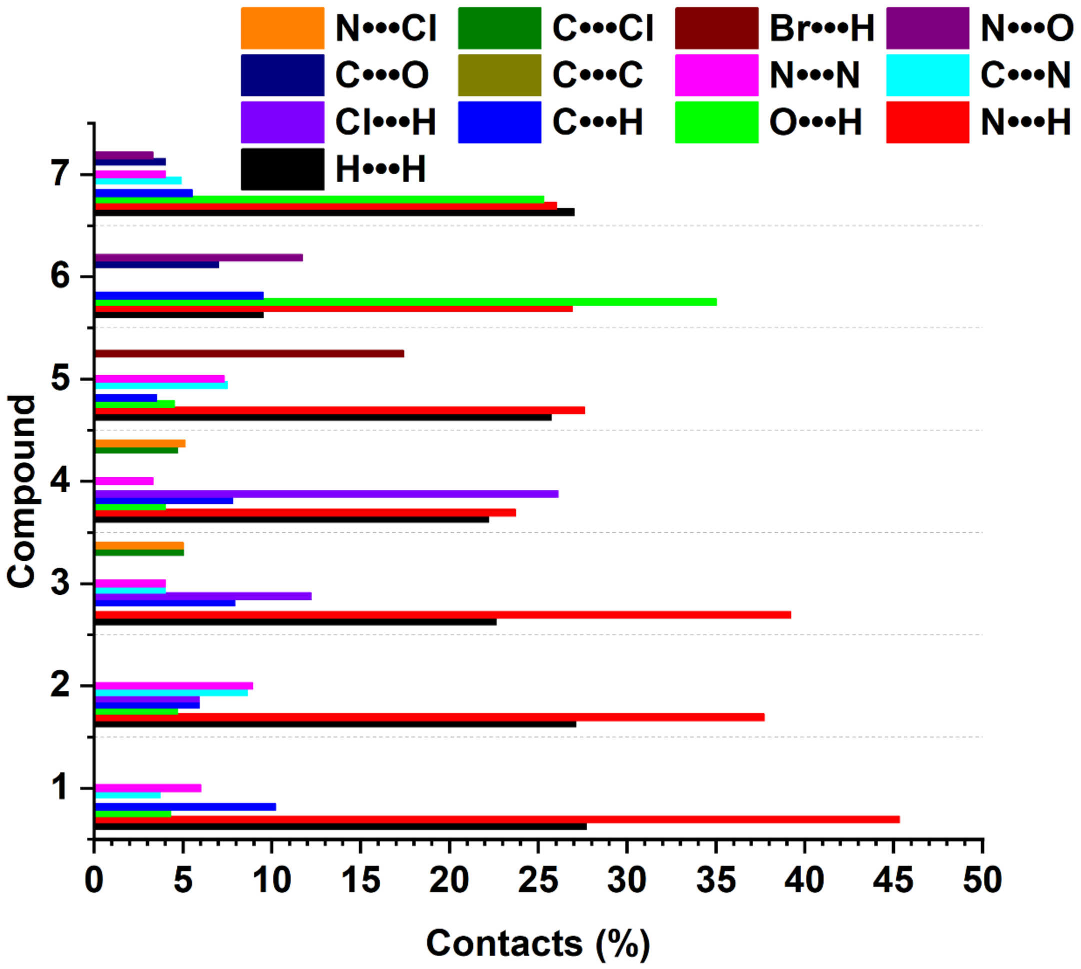

Figure S23. Histogram of contacts for compounds **1**-**7**.

6. Computational analysis of tautomers

6.1 Computational Details

Quantum chemical computations were carried with the Gaussian 16 package by using density functional theory (DFT).[15] The B3LYP functional was employed throughout in conjunction with the 6-31+G** basis set. B3LYP has proven to give excellent performance, nearly reproducing experimental electrical and optical properties for organic molecules.[1,16,17] Solvent (water) effects were included by the polarizable continuum model (PCM).[18] The nature of located stationary points was verified by checking the eigenvalues of the Hessian matrix; all the minimum energy structures have positive eigenvalues.

6.2 Discussion

We have performed a computational analysis of the energy of the tautomers of TTT1 in the neutral, monoprotonated, deprotonated and anionic forms; the results are reported in Table S4, while in Chart S1 is reported, for easy reading, one tautomer for each form, with indication of the adopted numbering of N atoms. The full list is given in Chart 2 of the typescript.

Chart S1**.** Chemical diagrams of one tautomer for each form of TTT1 with indication of numbering of N atoms.

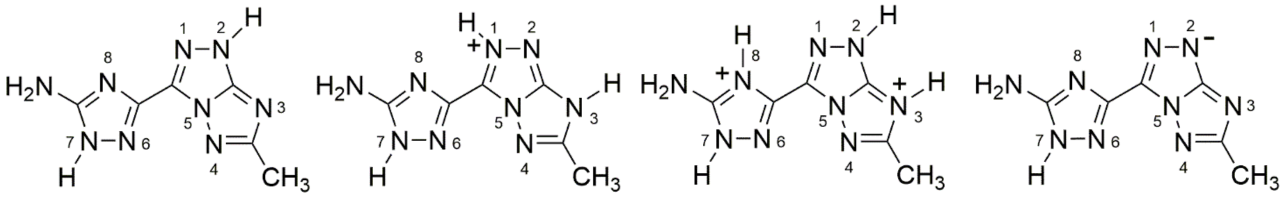

2H-7H   *1H-3H-7H*   *2H-3H-7H-8H*   *7H*

Table S4. Predicted relative energies (kcal/mol) of the possible tautomers of neutral, cationic and anionic species of TTT1, with the most stable tautomer highlighted in bold.

Tautomer	gas	water
2H-7H	**0.0**	**0.0**
2H-8H	3.58	4.56
3H-7H	8.37	2.47
3H-8H	10.5	6.27
4H-7H	14.6	9.45
4H-8H	15.8	18.0
1H-2H-7H	18.7	14.5
2H-3H-7H	8.68	1.69
2H-4H-7H	7.15	5.86

2H-6H-7H	14.2	8.68
2H-7H-8H	9.51	1.98
1H-3H-7H	**0.0**	**0.0**
3H-7H-8H	9.74	1.37
2H-3H-7H-8H	**0.0**	**0.0**
1H-3H-7H-8H	4.09	1.95
Anion 7H	1.58	**0.0**
Anion 2H	**0.0**	3.87

[a] The most stable tautomer is highlighted in bold.

The relative energies of tautomers are significantly different in gas and in polar medium, but the most stable species is predicted to be the same in both media except for the anions, where 2H is predicted to be the most stable tautomer in gas phase, while in polar medium it has a significantly higher energy than 7H.

The species which are predicted to be the most stable in polar medium are the ones which are experimentally observed, except for the monoprotonated one. Indeed, the 1H-3H-7H is predicted to be the lowest-energy species from our calculation, while the 3H-7H-8H and 2H-7H-8H are the species observed experimentally, which are predicted, respectively, to be 1.37 kcal/mol and 1.98 Kcal/mol higher in energy than 1H-3H-7H from our calculations.

We have also computed the relative energies for the conformers of each tautomer, Tab. S5, finding that only two different conformers are possible, differing for the relative orientation of the triamine rings, as depicted in Chart S2 below. The conformers are indicated by the letter *c* or *t*.

Chart S2. Definition of the Conformers for TTT1

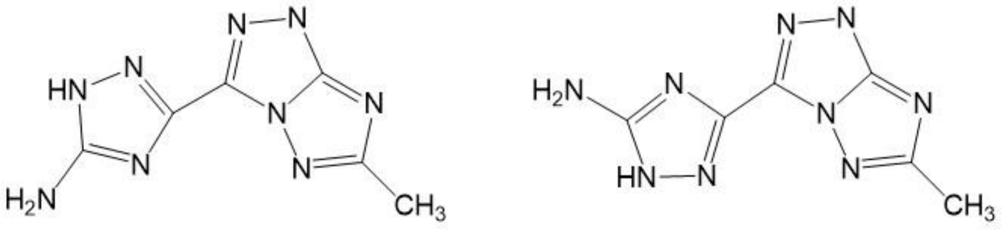

c **t**

Table S5. Predicted relative energies (kcal/mol) of the possible conformers of neutral, cationic, dicationic and monoanionic species in Fig. 1[a].

Tautomer	gas		water	
	c	*t*	*c*	*t*
2H-7H	0.75	**0.0**	0.85	**0.0**
2H-8H	3.58	5.43	4.91	4.57
3H-7H	8.90	8.37	3.13	2.47
3H-8H	14.6	10.5	7.38	6.28
4H-7H	14.8	14.6	9.49	9.45
4H-8H	15.8 [b]	15.8	18.7	18.0
1H-2H-7H	20.4	18.7	15.6	14.5
2H-3H-7H	9.33	8.68	2.41	1.69
2H-4H-7H	7.15	7.32	5.86	5.91
2H-6H-7H	17.8	14.2	9.28	8.68
2H-7H-8H	9.51	12.4	2.43	1.98
1H-3H-7H	1.24	**0.0**	0.94	**0.0**
3H-7H-8H	14.3	9.74	2.72	1.38
2H-3H-7H-8H	3.58	**0.0**	1.31	**0.0**
1H-3H-7H-8H	4.09	11.3	1.95	3.64
Anion 2H	2.34	1.58	0.82	**0.0**
Anion 7H	0.48	**0.0**	4.59	3.86

[a] The most stable conformation is highlighted in bold.
[b] Interconverts in conformer *t* after geometry optimization.

In gas phase, the conformation *t* is predicted to be the most stable one for all tautomers except for the monoprotonated 2H-4H-7H, probably because in this species the conformer *c* is stabilized by intramolecular hydrogen bonds between the H in position 4 and the NH_2 group (see Chart S1). Also, the diprotonated species 1H-3H-7H-8H is predicted to be more stable in its *c* form, because of the steric hindrance between 1H and 8H in the *t* form.

Very similar energetic trends are found in water, even though the relative energy difference between conformers is significantly lower than in gas phase, except for monoprotonated 1H-2H-7H and 2H-4H-7H, whose conformers show relative energy difference almost identical in both media.

7. Energetic performances of 6

The density at room temperature of 1.69 g/cm^3 was obtained from the density of the low-temperature crystal structure and re-calculated to room temperature according to ref. 19. The enthalpy of formation was calculated applying the atomization method (refs 15 and 19) and estimating the lattice enthalpies according to the method of Gutowski (ref. 19) which is based on VBT (volume-based thermodynamics). The detonation parameters were calculated using the EXPLO5 code.[20]

Table S6. Energetic performances of **6**.

formula	$C_6H_8N_9ClO_4$
M / g mol^{-1}	305.6
density (173 K, exptl.) / g cm^{-3}	1.722
density (298 K, recalcd.)[19] / g cm^{-3}	1.69
ΔU_L (Gutowski)[19] / kJ mol^{-1}	-563.3
ΔH_L (Gutowski)[19] / kJ mol^{-1}	-568.3
CBS-QB3 enthalpy[15,19] (298 K) / H	H_3L^+ -724.821377<br>ClO_4^- -760.151815
$\Delta H°_f$ (g) (calcd.)[19] / kJ mol^{-1}	$HT3^+$ +1162.4<br>ClO_4^- -271.6
$\Delta H°_f$ (s) (calcd.)[19] kJ mol^{-1}	+322.5
$\Omega(CO_2)$ / % (oxygen balance based on CO_2)	-60.2
$-Q_{ex}$ / kJ kg^{-1} (heat of explosion)[20]	3820
T_{ex} / K [20]	2958
p_{C-J} / GPa (detonation pressure)[20]	19.6
VoD / m s^{-1} (detonation velocity)[20]	7158
V_0 / dm^3 kg^{-1} (volume of explosion gases)[20]	686
TNT_{eq} (from Q_{ex})[20] / %	87.6
impact sensitivity / J	15 (sensitive)
friction sensitivity / N	> 360 (insensitive)
ESD / J	1.0

8. High Pressure Computations

8.1 Computational Details

Periodic density functional theory (DFT) calculations were performed using the Vienna Ab Initio Simulation Package (VASP).[21,22] The Generalized Gradient Approximation functional of Perdew, Burke, and Ernzerhof (GGA-PBE)[23] was used in combination with the projector augmented wave (PAW) method to describe electron–ion interactions. Van der Waals dispersion forces were treated using the DFT-D3 method with Becke–Johnson damping.[24,25,26]

A plane-wave energy cutoff of 520 eV and an electronic convergence criterion of 10^{-6} eV were applied throughout. The Brillouin zone was sampled using a Γ-centered 3x4x2 k-point grid, selected for consistency with the monoclinic lattice dimensions of **6**. Full relaxation of atomic positions, cell shape, and cell volume was performed at each pressure point. All calculations were non-spin polarized, consistent with the non-magnetic character of the system. Gaussian smearing with a width of 0.05 eV was used for both structures.

The actual (3H-7H-8H) and virtual (1H-3H-7H) structures of **6** were used as the initial configurations. The virtual structure was generated by shifting the hydrogen atom from N8 to N1 in the actual structure without any other modification, as described in the main text. Both structures were first relaxed at zero external pressure, Fig. S24, to validate the method: the computed lattice parameters are in close agreement with the experimental crystal structure of **6**, confirming that the DFT-D3 approach accurately captures the structural properties of this system. The perchlorate anions (ClO_4^-) maintain identical positions in both structures throughout the calculations.

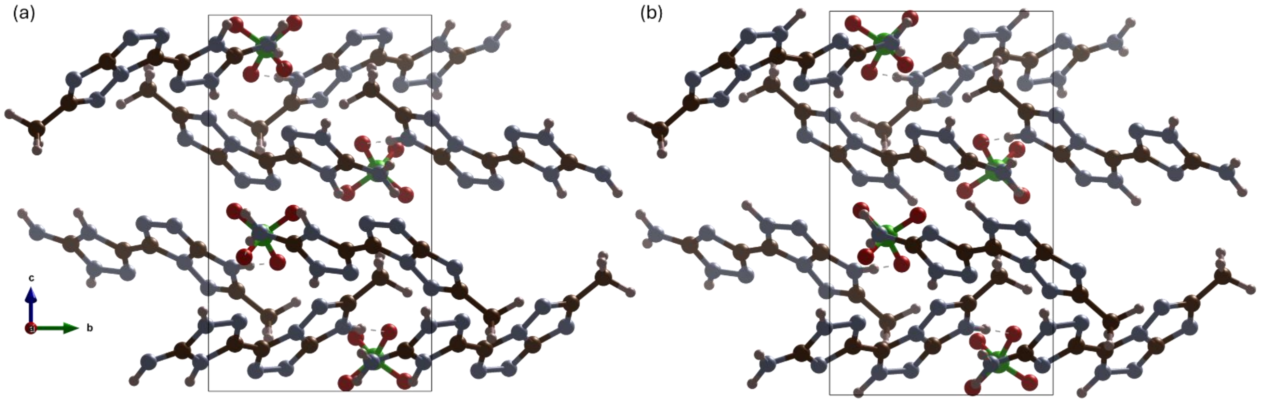

Figure S24. Optimized structures of (a) actual and (b) virtual configuration of **6**, showing H atom position at N8 vs N1.

8.2 Additional Results

8.2.1 Pressure series and dynamic instability of the virtual structure.

Geometry optimizations were performed at the following pressures: 0.1 MPa, 0.4 GPa, 0.6 GPa, 1.2 GPa, 2.0 GPa, 3.0 GPa, 4.3 GPa, 5.2 GPa, 6.4 GPa, 7.1 GPa, 8.4 GPa, and 9.3 GPa. The actual (3H-7H-8H) structure is successfully optimized at all pressures and corresponds to a stable minimum of the lattice energy throughout the full pressure range investigated. The virtual (1H-3H-7H) structure is successfully optimized up to 2.0 GPa, but initial calculations at 3.0 GPa revealed spontaneous conversion to the 3H-7H-8H configuration during geometry relaxation. To precisely identify the instability threshold, additional calculations were performed at intermediate pressures between 2.0 and 3.0 GPa. These systematic calculations identify ~2.8 GPa as the critical pressure beyond which the 1H-3H-7H structure no longer occupies a local minimum on the potential energy surface. Beyond this threshold, DFT geometry optimizations initialized from the 1H-3H-7H configuration spontaneously converge to the 3H-7H-8H structure during relaxation, regardless of starting geometry or optimization parameters. This critical pressure corresponds to a unit cell volume of approximately 1025 $Å^3$ for the virtual structure.

This behavior reflects a fundamental loss of metastability rather than a thermodynamic phase transition. The local potential energy minimum sustaining the 1H-3H-7H form simply ceases to exist under compression. As pressure increases, compression of the lattice shortens intermolecular distances, making the 1H-3H-7H hydrogen-bonding network increasingly unfavorable relative to the 3H-7H-8H arrangement. The energy barrier separating the two tautomeric forms progressively decreases until it vanishes entirely beyond ~2.8 GPa, at which point no computationally stable 1H-3H-7H configuration can exist.

8.2.2. Lattice parameter evolution under pressure.

At ambient conditions, the optimized 3H-7H-8H structure exhibits lattice parameters a = 11.22 Å, b = 7.89 Å, c = 15.54 Å, and β = 121.17°, with a volume of 1177.8 $Å^3$. The virtual 1H-3H-7H structure shows nearly identical values: a = 11.07 Å, b = 7.87 Å, c = 15.60 Å, and β = 120.11°, with a volume of 1174.8 $Å^3$. The near-identical ambient lattice parameters of the two structures reflect the fact that they differ only in the position of a single hydrogen atom, with the same overall crystal packing arrangement and space group.

Both structures compress comparably at low pressure. At 2.0 GPa, the 3H-7H-8H structure shows a

= 10.70 Å, b = 7.72 Å, c = 14.58 Å, β = 117.96°, V = 1064.2 Å³, and the 1H-3H-7H structure shows a = 10.10 Å, b = 7.76 Å, c = 14.50 Å, β = 113.55°, V = 1042.5 Å³. Volume reductions from ambient to 2.0 GPa are 9.6% for the actual and 11.3% for the virtual structure. The slightly greater compression of the virtual structure is consistent with its lower bulk modulus (12.14 GPa vs. 13.36 GPa), reflecting weaker intermolecular interactions in the 1H-3H-7H hydrogen-bonding network.

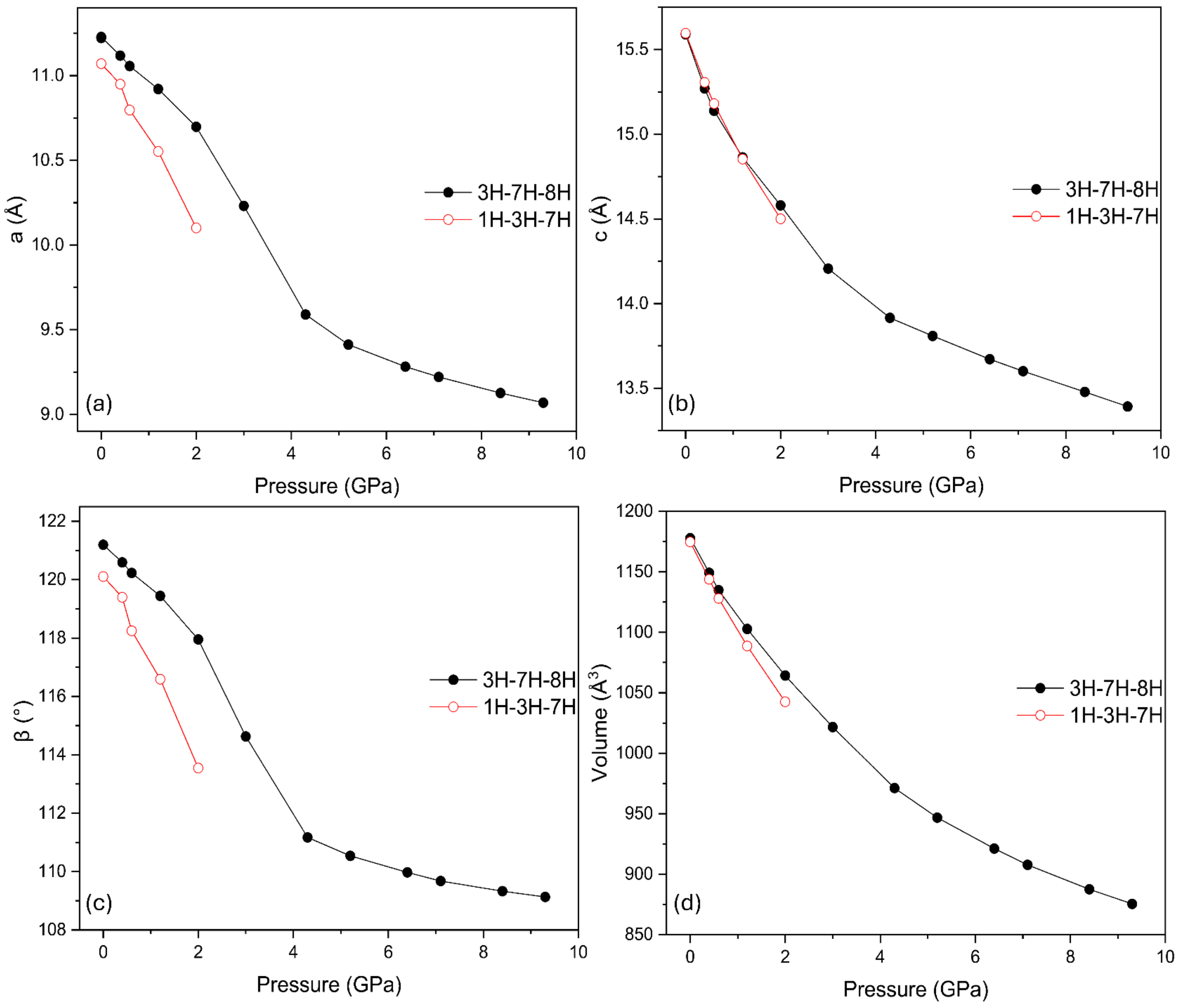

Figure S25. Calculated lattice parameters of the 3H-7H-8H and 1H-3H-7H crystal structures of **6** vs pressure. The 1H-3H-7H tautomer structure is shown only where stable (0 – 2.0 GPa). (a) a-axis; (b) c-axis; (c) β angle; (d) unit cell volume.

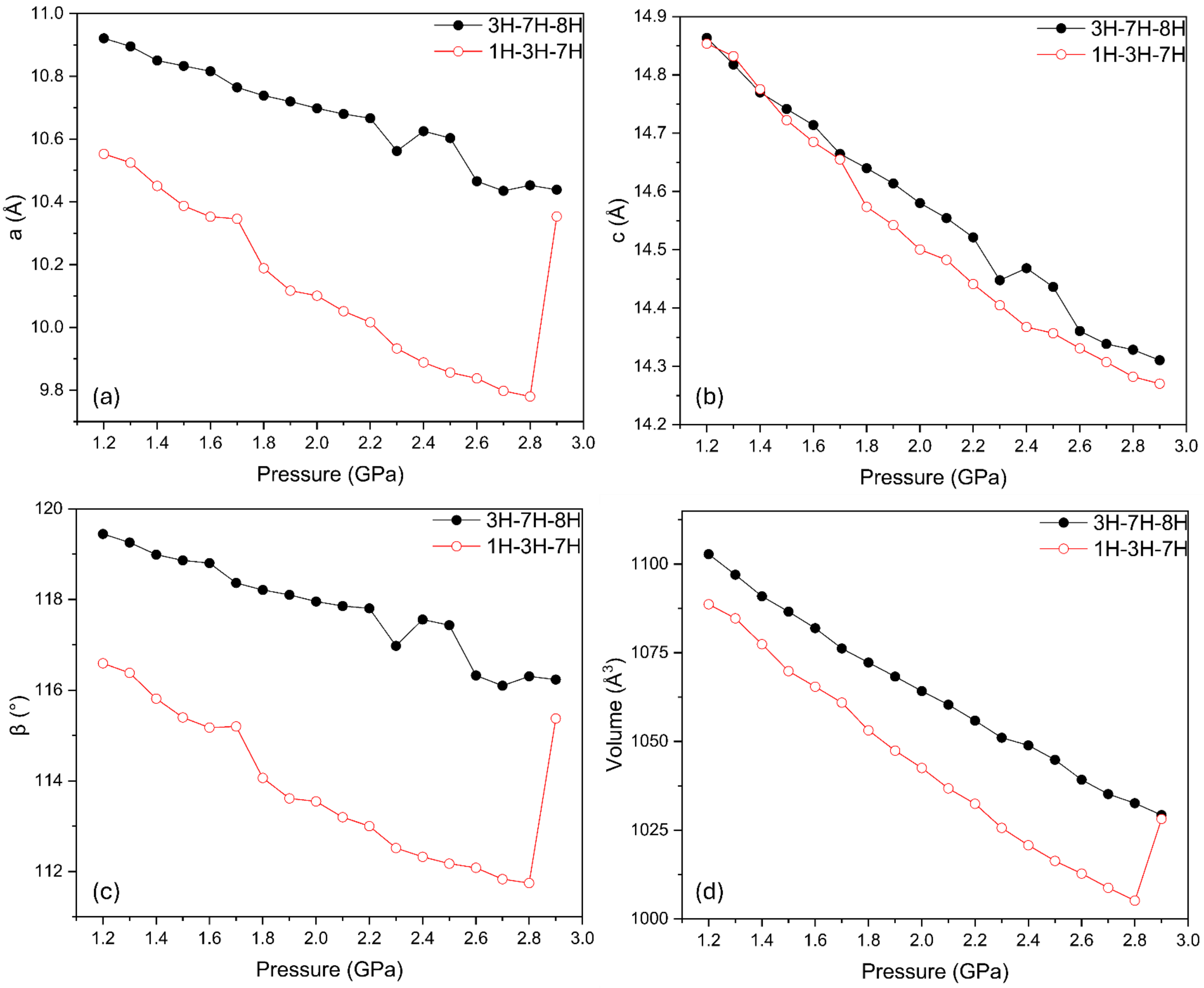

Figure S26. Calculated lattice parameters of the actual and virtual structures of **6** vs pressure in the 1.2–3.0 GPa range. (a) a-axis; (b) c-axis; (c) β angle; (d) unit cell volume V. The anomalous upturn in the virtual structure at 2.9 GPa reflects spontaneous conversion to the 3H-7H-8H configuration during geometry optimization, marking the loss of the 1H-3H-7H local minimum beyond ~2.8 GPa.

For the 3H-7H-8H tautomer, which remains the sole stable configuration beyond 2.0 GPa, compression continues across the extended pressure range. The a unit cell parameter undergoes a total reduction of 19.2% from ambient, decreasing from 10.70 Å at 2.0 GPa to 10.23 Å at 3.0 GPa, 9.59 Å at 4.3 GPa, and 9.07 Å at 9.3 GPa. The b parameter is the most resistant to compression, showing a total reduction of only 3.3% from ambient conditions (7.89 → 7.63 Å at 9.3 GPa). The c parameter compresses smoothly and monotonically, decreasing 6.2% from ambient to 2.0 GPa and 13.8% overall to 9.3 GPa (15.54 → 13.39 Å). The β-angle decreases systematically from 121.17° at ambient to 109.13° at 9.3 GPa, a total reduction of 12.04°. A particularly notable transition region is observed between 6.4 and 7.1 GPa, where coordinated changes in a, b, and β occur simultaneously, suggesting a region of enhanced structural reorganization. Full tabulated lattice parameters at each

pressure point are given in Table S7.

Table S7. Lattice parameters of the 3H-7H-8H tautomer and 1H-3H-7H tautomer.

	3H-7H-8H					1H-3H-7H				
Pressure (GPa)	a (Å)	b (Å)	c (Å)	β (°)	V (Å^3)	a (Å)	b (Å)	c (Å)	β (°)	V (Å^3)
0	11.22	7.89	15.54	121.2	1177.8	11.07	7.87	15.60	120.1	1174.8
0.4	11.12	7.86	15.27	120.6	1149.2	10.95	7.83	15.31	119.4	1143.8
0.6	11.06	7.85	15.14	120.2	1134.8	10.80	7.81	15.18	118.2	1128.0
1.2	10.92	7.80	14.86	119.4	1102.8	10.55	7.77	14.85	116.6	1088.7
1.3	10.90	7.79	14.82	119.3	1097.0	10.53	7.76	14.83	116.4	1084.7
1.4	10.85	7.78	14.77	119.0	1090.9	10.45	7.75	14.78	115.8	1077.4
1.5	10.83	7.77	14.74	118.9	1086.6	10.39	7.74	14.72	115.4	1069.8
1.6	10.82	7.76	14.71	118.8	1081.9	10.35	7.74	14.69	115.2	1065.4
1.7	10.77	7.75	14.67	118.4	1076.2	10.35	7.73	14.66	115.2	1061.0
1.8	10.74	7.74	14.64	118.2	1072.2	10.19	7.77	14.57	114.1	1053.1
1.9	10.72	7.73	14.61	118.1	1068.3	10.12	7.77	14.54	113.6	1047.4
2	10.70	7.72	14.58	118.0	1064.2	10.10	7.76	14.50	113.5	1042.5
2.1	10.68	7.72	14.55	117.9	1060.4	10.05	7.75	14.48	113.2	1036.8
2.2	10.67	7.71	14.52	117.8	1055.9	10.02	7.75	14.44	113.0	1032.5
2.3	10.56	7.73	14.45	117.0	1051.1	9.93	7.76	14.41	112.5	1025.7
2.4	10.63	7.70	14.47	117.6	1048.9	9.89	7.77	14.37	112.3	1020.8
2.5	10.60	7.69	14.44	117.4	1044.8	9.86	7.76	14.36	112.2	1016.3
2.6	10.47	7.72	14.36	116.3	1039.2	9.84	7.75	14.33	112.1	1012.8
2.7	10.44	7.70	14.34	116.1	1035.2	9.80	7.75	14.31	111.8	1008.7
2.8	10.45	7.69	14.33	116.3	1032.6	9.78	7.75	14.28	111.7	1005.1
2.9	10.44	7.68	14.31	116.2	1029.2					
3	10.23	7.73	14.21	114.6	1021.6					
4.3	9.59	7.78	13.92	111.2	971.2					
5.2	9.41	7.75	13.81	110.5	946.8					
6.4	9.28	7.72	13.67	110.0	921.2					
7.1	9.22	7.69	13.60	109.7	907.7					
8.4	9.13	7.65	13.48	109.3	887.5					

9.3	9.07	7.63	13.39	109.1	875.5					

The pronounced compression anisotropy (a: −19.2%, c: −13.8%, b: −3.3%, β: −10.0%) indicates that the crystal structure is most resistant to compression along the b-axis, with the a-axis and β-angle being most responsive to pressure. This behavior, characteristic of layered or chain-like molecular packing, is consistent with the experimental trends reported in Fig. 10 of the main text, including the experimentally observed drop in β above 1.2 GPa, which is correctly reproduced by the calculations.

8.2.3 Equation of state analysis

The energy-volume data for both structures were fitted to the Vinet equation of state (EOS), which provides an accurate description of the compression behavior of molecular crystals. Results are summarized in Table S8.

Table S8. Vinet EOS parameters for actual and virtual structures of **6**.

	3H-7H-8H (actual)	1H-3H-7H (virtual)
V_0 (Å^3)	1185.4	1176.1
E_0 (eV)	−729.46	−728.67
B_0 (GPa)	13.36	12.14
B_0'	5.07	4.54
Pressure range fitted	0 – 9.3 GPa	0 – 2.0 GPa

For the actual (3H-7H-8H) structure, fitting across the full pressure range (13 data points, 0.1 MPa to 9.3 GPa) yields: V_0 = 1185.4 Å^3, E_0 = −729.46 eV, B_0 = 13.36 GPa, B_0' = 5.07. For the virtual (1H-3H-7H) structure, fitting within the metastable regime (6 data points, 0.1 MPa to 2.0 GPa) yields: V_0 = 1176.1 Å^3, E_0 = −728.67 eV, B_0 = 12.14 GPa, B_0' = 4.54.

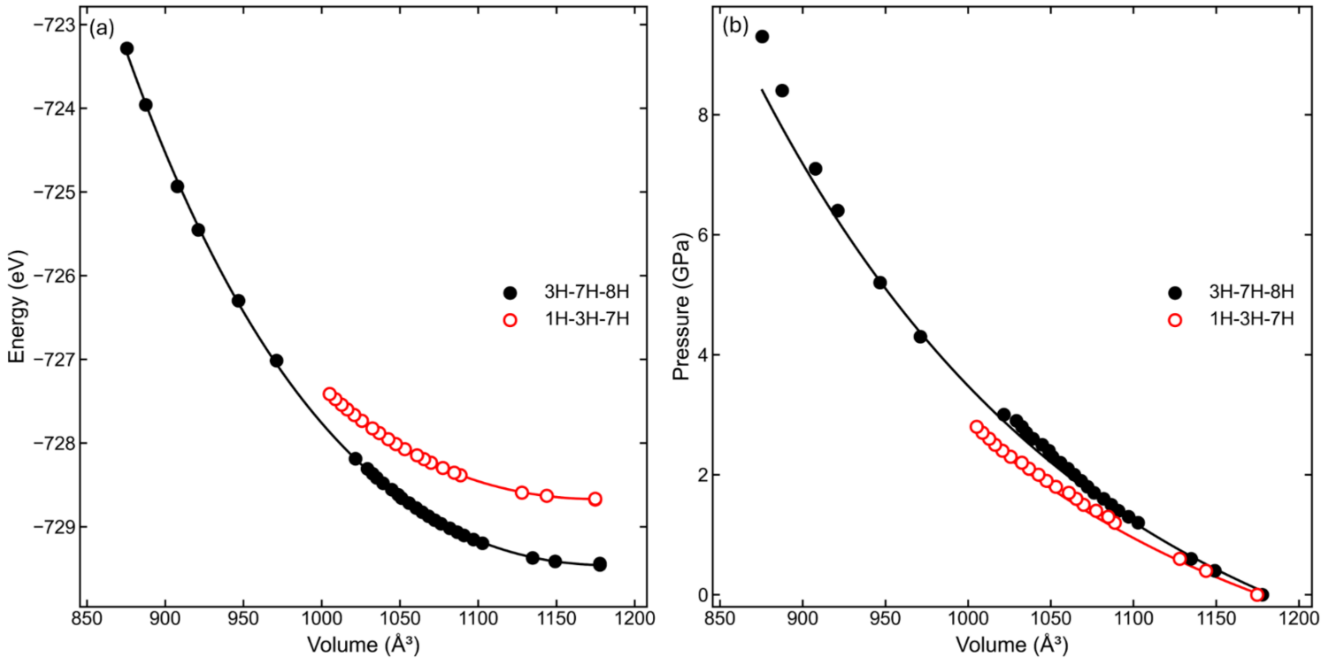

Figure S27. Equation of state for the actual and virtual structures of **6**. (a) Energy-volume and (b) pressure-volume curves. Solid lines are Vinet EOS fits. The virtual structure is shown only within its metastable regime, terminating at ~2.8 GPa (~1025 Å³) beyond which the 1H-3H-7H local minimum ceases to exist.

The actual structure is approximately 10% stiffer than the virtual structure (B_0 = 13.36 vs. 12.14 GPa), reflecting more favorable intermolecular packing and a stronger hydrogen-bonding network in the 3H-7H-8H form. The larger B_0' of the actual structure (5.07 vs. 4.54) indicates that its bulk modulus increases more rapidly with pressure, consistent with a more mechanically robust crystal structure. Both structures show similar compression ratios in the low-pressure regime (1.11 for 3H-7H-8H and 1.13 for 1H-3H-7H from ambient to 2.0 GPa), demonstrating comparable compressibility despite different bulk moduli over this limited pressure range. The actual structure compresses smoothly from 1177.8 Å³ at ambient to 875.5 Å³ at 9.3 GPa with no discontinuities, confirming the absence of phase transitions across the full range investigated.

8.2.4. Molecular-level bond length analysis.

To isolate tautomeric effects from compression effects, an isovolumetric comparison was performed between the virtual (1H-3H-7H) structure at 2.2 GPa (V = 1032.5 Å³) and the actual (3H-7H-8H) structure at 2.8 GPa (V = 1032.6 Å³). Selecting structures at different pressures but matched by volume allows direct comparison of the two tautomeric forms independent of compression state.

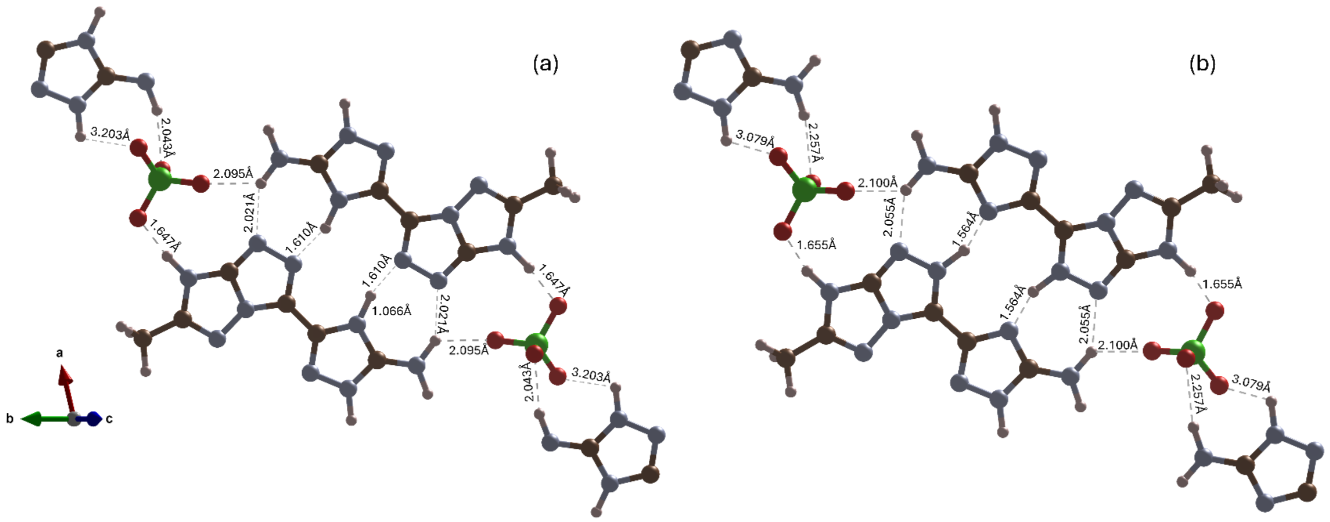

Figure S28. Bond length comparison for (a) 3H-7H-8H tautomer at 2.8 GPa and (b) 1H-3H-7H tautomer at 2.2 GPa.

All intramolecular covalent bonds differ by less than 1.5% between the two isovolumetric structures, confirming that the dynamic instability of the virtual structure does not arise from excessive bond strain; the molecular framework remains intact. Instead, large atomic displacements of 0.3–0.6 Å between the two tautomeric forms reflect fundamentally different hydrogen-bonding networks and intermolecular packing arrangements. As the virtual structure is compressed beyond 2.2 GPa toward the instability threshold at ~2.8 GPa, its weaker intermolecular forces cannot sustain the increasing energetic cost of unfavorable packing as intermolecular distances decrease, leading to spontaneous collapse to the globally stable 3H-7H-8H configuration. This demonstrates that molecular-level structural resilience (intact covalent geometry) does not guarantee macroscopic crystal stability; failure arises at the intermolecular rather than the intramolecular scale.

8.2.5. Thermodynamic analysis and enthalpy comparison.

The relative lattice enthalpy $\Delta H = H(\text{virtual}) - H(\text{actual})$ was calculated at each pressure where both structures exist as distinct configurations, using $H = E + PV$ with E from the DFT total energies and V from the optimized unit cell volumes.

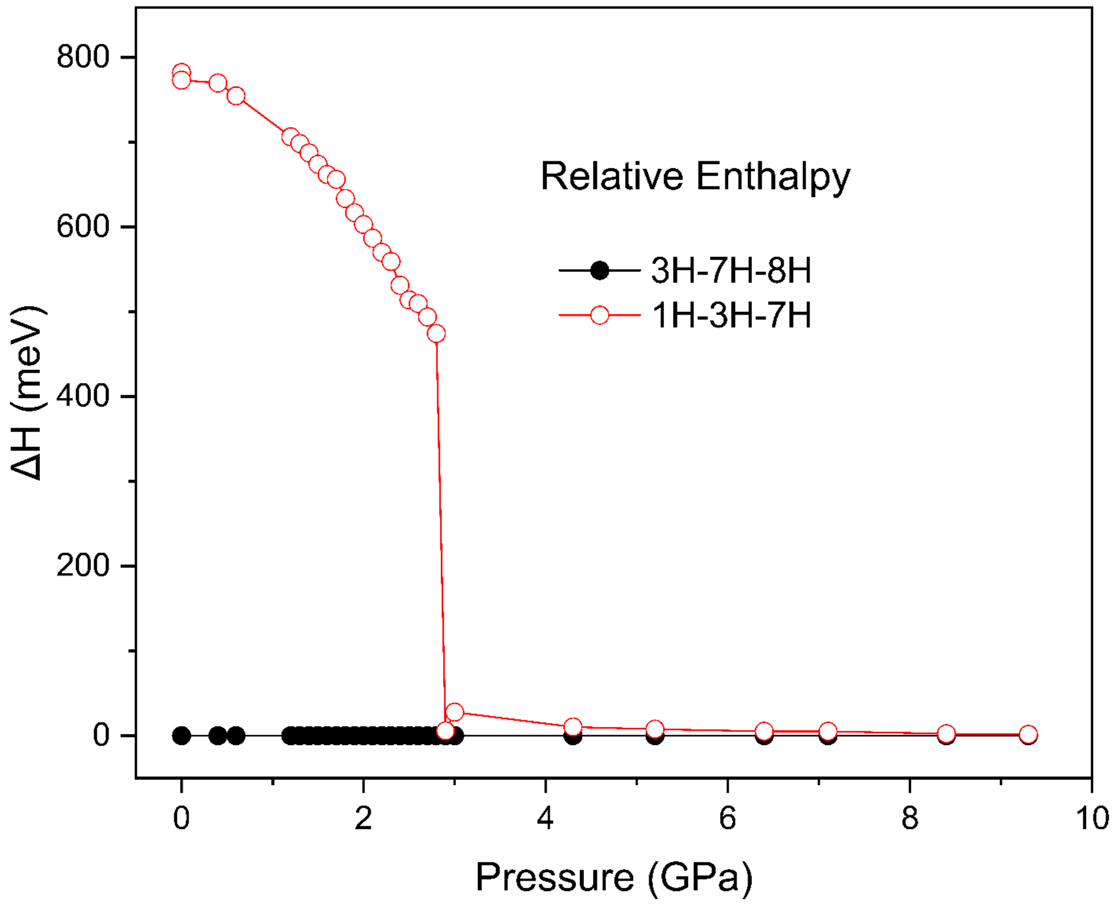

Figure S29. Relative enthalpy ΔH = H(1H-3H-7H) – H(3H-7H-8H) vs. pressure for **6**. ΔH decreases from 798 meV at ambient to 403 meV at 2.0 GPa. Beyond ~2.8 GPa, both calculations converge to the 3H-7H-8H structure; ΔH → 0 is a computational artifact, not a thermodynamic crossover.

At ambient pressure, the virtual structure is higher in enthalpy by 798 meV (0.80 eV, or 18.4 kcal/mol on a per-formula-unit basis), confirming that the actual 3H-7H-8H structure is thermodynamically preferred. The enthalpy difference decreases systematically with pressure: 798 meV at 0.1 MPa, 602 meV at 1.2 GPa, and 403 meV at 2.0 GPa. This trend reflects partial compensation of the 1H-3H-7H form's intrinsic energy disadvantage by the PV term as pressure increases. Nonetheless, ΔH remains positive throughout the entire metastable regime, confirming that the actual structure is thermodynamically preferred at all pressures where both configurations exist.

Above ~2.8 GPa, geometry optimizations initialized from the virtual configuration converge to the actual 3H-7H-8H structure. Consequently, enthalpy values labeled as "virtual" at higher pressures in fact describe the actual structure optimized from a different starting geometry, and the apparent decrease of ΔH toward zero at high pressure is a computational artifact rather than a true thermodynamic crossover. This convergence is itself confirmation that the 1H-3H-7H local energy minimum has disappeared. The data in this region (shown in Fig. 12 of the main text and in Fig. S29

above) should not be interpreted as evidence of thermodynamic equivalence between the two crystal structures (actual and virtual).

In the static lattice (0 K) framework, the 3H-7H-8H tautomer is therefore the thermodynamically stable form at all pressures investigated. This picture is fully consistent with the crystal growth hypothesis discussed in the main text, in which cations enter the pre-nucleation cluster as the most abundant solution-phase 1H-3H-7H tautomer and then rapidly switch by proton transfer to the more stable 3H-7H-8H dimer arrangement without disrupting the growing crystal structure.

References

1.Parisi, E.; Landi, A.; Fusco, S.; Manfredi, C.; Peluso, A.; Wahler, S.; Klapötke, T. M.; Centore R. High-Energy-Density Materials: An Amphoteric N-Rich Bis(triazole) and Salts of Its Cationic and Anionic Species. *Inorg. Chem.* **2021**, *60*, 16213-16222.

2.Biedermann G.; Sillén, L. G. Studies on the hydrolysis of metal ions. IV. *Ark. Kemi.* **1953**, *40*, 425-440.

3.Gran, G. Determination of the equivalence point in potentiometric titrations. Part II. *Analyst* **1952**, *77*, 661–671.

4.Rossotti, F. J. C.; Rossotti, H. S. *The Determination of Stability Constants and Other Equilibrium Constants in Solution*, McGraw-Hill, New York, 1961.

5.Gans, P.; Sabatini, A.; Vacca, A. Investigation of equilibria in solution. Determination of equilibrium constants with the HYPERQUAD suite of programs. *Talanta* **1996**, *43*, 1739-1753.

6.Bruker-Nonius (2002) SADABS, Bruker-Nonius, Delft, The Netherlands.

7.Altomare, A.; Burla, M. C.; Camalli, M.; Cascarano, G. L.; Giacovazzo, C.; Guagliardi, A.; Moliterni, G. G.; Polidori, G.; Spagna R. SIR97: a new tool for crystal structure determination and refinement. *J. Appl. Crystallogr.* **1999**, *32*, 115-119.

8.Sheldrick, G. M. Crystal structure refinement with SHELXL. *Acta Crystallogr.* **2015**, *C71*, 3-8.

9.Farrugia, L. J. WinGX and Ortep for Windows: an update. *J. Appl. Crystallogr.* **2012**, *45*, 849-854.

10.Macrae, C. F.; Bruno, I. J.; Chisholm, J. A.; Edgington, P. R.; McCabe, P.; Pidcock, E.; Rodriguez-Monge, L.; Taylor, R.; van de Streek, J.; Wood, P. A. Mercury CSD 2.0 – new features for the visualization and investigation of crystal structures. *J. Appl. Cryst.* **2008**, *41*, 466-470.

11. Mao, H. K.; Xu, J.; Bell, P. M. Calibration of the Ruby Pressure Gauge to 800 kbar under Quasi-hydrostatic Conditions. *J. Geophys. Res.* **1986**, *91*, 4673– 4676.

12. Xcalibur CCD System, CrysAlisPro Software System, ver. 1.171.33; Oxford Diffraction Ltd.: Wrocław, Poland, 2009.

13.Spackman, P. R.; Turner, M. J.; McKinnon, J. J.; Wolff, S. K.; Grimwood, D. J.; Jayatilaka, D.; Spackman, M. A. CrystalExplorer: a program for Hirshfeld surface analysis, visualization and quantitative analysis of molecular crystals. *J. Appl. Cryst.* **2021**, *54*, 1006–1011.

14.Spackman, M. A.; McKinnon, J. J. Fingerprinting intermolecular interactions in molecular crystals. *CrystEngComm* **2002**, *4*, 378–392.

15. M. J. Frisch, G. W. Trucks, H. B. Schlegel, G. E. Scuseria, M. A. Robb, J. R. Cheeseman, G. Scalmani, V. Barone, G. A. Petersson, H. Nakatsuji, X. Li, M. Caricato, A. V. Marenich, J. Bloino, B. G. Janesko,

R. Gomperts, B. Mennucci, H. P. Hratchian, J. V. Ortiz, A. F. Izmaylov, J. L. Sonnenberg, D. Williams-Young, F. Ding, F. Lipparini, F. Egidi, J. Goings, B. Peng, A. Petrone, T. Henderson, D. Ranasinghe, V. G. Zakrzewski, J. Gao, N. Rega, G. Zheng, W. Liang, M. Hada, M. Ehara, K. Toyota, R. Fukuda, J. Hasegawa, M. Ishida, T. Nakajima, Y. Honda, O. Kitao, H. Nakai, T. Vreven, K. Throssell, J. A. Montgomery Jr., J. E. Peralta, F. Ogliaro, M. J. Bearpark, J. J. Heyd, E. N. Brothers, K. N. Kudin, V. N. Staroverov, T. A. Keith, R. Kobayashi, J. Normand, K. Raghavachari, A. P. Rendell, J. C. Burant, S. S. Iyengar, J. Tomasi, M. Cossi, J. M. Millam, M. Klene, C. Adamo, R. Cammi, J. W. Ochterski, R. L. Martin, K. L. Morokuma, O. Farkas, J. B. Foresman, D. J. Fox, Gaussian 16 Revision C.01, Gaussian Inc., Wallingford CT, USA 2016.

16. Capobianco, A.; Borrelli, R.; Landi, A.; Velardo, A.; Peluso, A. Absorption Band Shapes of a Push-Pull Dye Approaching the Cyanine Limit: A Challenging Case for First Principle Calculations. *J. Phys. Chem. A* **2016**, *120*, 5581−5589.

17. Landi, A.; Borrelli, R.; Capobianco, A.; Velardo, A.; Peluso, A. Second-Order Cumulant Approach for the Evaluation of Anisotropic Hole Mobility in Organic Semiconductors. *J. Phys. Chem. C* **2018**, *122*, 25849−25857.

18. Miertuš, S.; Scrocco, E.; Tomasi, J. Electrostatic interaction of a solute with a continuum. A direct utilizaion of AB initio molecular potentials for the prevision of solvent effects. *Chem. Phys.* **1981**, *55*, 117−129.

19. Klapötke, T. M. *Chemistry of High Energy Materials*, 7th edn., Walter de Gruyter, Berlin/Boston, **2025**.

20. EXPLO5_V8.01.01, M. Suceska, Zagreb, **2024**.

21. Kresse G.; Hafner, J. Ab initio molecular dynamics for liquid metals. *Phys. Rev. B* **1993**, *47*, 558-561.

22. Kresse, G.; Hafner, J. Ab initio molecular-dynamics simulation of the liquid-metal--amorphous-semiconductor transition in germanium. *Phys. Rev. B* **1994**, 49, 14251-14269.

23. Perdew, J. P.; Burke, K.; Ernzerhof, M. Generalized gradient approximation made simple. *Phys. Rev. Lett.* **1996**, *77*, 3865-3868.

24. Grimme, S.; Antony, J.; Ehrlich, S.; Krieg, H. A consistent and accurate ab initio parametrization of density functional dispersion correction (DFT-D) for the 94 elements H-Pu. *J. Chem. Phys.* **2010**, *132*, 154104.

25. Grimme, S.; Ehrlich, S.; Goerigk, L. Effect of the damping function in dispersion corrected density functional theory. *J. Comput. Chem.* **2011**, *32*, 1456-1465.

26. Goerigk, L. A Comprehensive Overview of the DFT-D3 London-Dispersion Correction, in Non-Covalent Interactions in Quantum Chemistry and Physics, Chapter 6, edited by A. Otero de la Roza and G. A. DiLabio (Elsevier, 2017), pp. 195–219.
